# Validating the neurite exchange imaging (NEXI) model of gray matter using Monte Carlo simulations in numerical substrates

**DOI:** 10.1162/IMAG.a.1381

**Published:** 2026-10-06

**Authors:** Rita Oliveira, Jasmine Nguyen-Duc, Malte Brammerloh, Ileana Jelescu

**Affiliations:** Department of Radiology, Lausanne University Hospital (CHUV) and University of Lausanne, Lausanne, Switzerland

**Keywords:** diffusion MRI, NEXI, biophysical model, numerical substrates

## Abstract

NEXI is a gray matter (GM) microstructural model designed to probe brain tissue microstructure *in vivo* using diffusion MRI. NEXI describes GM as two exchanging Gaussian compartments—neurites, modeled as randomly oriented, infinitely long sticks, and the extracellular space—allowing the estimation of biophysically interpretable parameters related to neurite microstructure and intercompartmental exchange. While modeling cell processes as sticks and each compartment as Gaussian are common assumptions for brain biophysical models of diffusion, neurite structural irregularities and the presence of somas, particularly in GM, may violate them and bias NEXI parameter estimates. Furthermore, the barrier-limited exchange assumed in the Kärger model that underlies NEXI may also be violated in realistic conditions. Therefore, in this work, we evaluate NEXI’s accuracy in numerical substrates that contain GM features such as neurite beading, undulation, orientation dispersion, and the presence of somas across a range of membrane permeabilities. Diffusion signals were generated with Monte Carlo simulations of water diffusion and subsequently fitted with NEXI. Overall, NEXI captured trends of exchange time across permeability levels and showed good ability to disentangle exchange effects from other microstructural features. The largest bias in *t_ex_* was introduced by somas, and in *D_i_* by permeability, somas and beading. Otherwise, the structural irregularities evaluated in this study had a relatively modest impact on NEXI estimates. Most bias in NEXI estimates comes from finite SNR. Future work should aim to further improve substrate realism to better represent the complexity of GM morphology.

## 1 Introduction

Characterization of brain tissue microstructure *in vivo* from Magnetic Resonance Imaging (MRI) has long been a major focus in neuroscience, as it enables non-invasive investigation of the structural underpinnings of brain function in health and disease (Novikov et al., 2018; Weiskopf et al., 2015, 2021). Diffusion MRI, in particular, is a powerful tool for this purpose, as the signal reflects the motion of water molecules and the constraints imposed by the underlying tissue architecture. However, because diffusion MRI acquisitions have a millimeter-scale resolution, they cannot directly resolve microscopic features of brain tissue, such as cell size. To bridge this gap, biophysical models link the diffusion MRI signal to parameters that describe the underlying tissue in a simplified geometry. This non-invasive approach, often referred to as *Microstructural MRI* (Jelescu et al., 2020; Novikov et al., 2018; Weiskopf et al., 2015), has become an important framework for probing brain tissue microstructure *in vivo*.

Within this framework, several biophysical models have been proposed, each relying on different assumptions and tailored to specific tissue types. In white matter (WM), models such as the Standard Model (SM) (Coelho et al., 2024; Novikov et al., 2018) have been widely adopted. In gray matter (GM), commonly used models include the Soma and Neurite Density Imaging (SANDI), which extends the SM by adding a spherical compartment to represent cell bodies (Palombo et al., 2020), and Neurite Exchange Imaging (NEXI) (Jelescu et al., 2022) or Standard Model with Exchange (SMEX) (Olesen et al., 2022), which includes water exchange between cell processes (dendrites, axons, and glial processes) and the extracellular space.

More specifically, the NEXI/SMEX (Jelescu et al., 2022; Olesen et al., 2022; Uhl et al., 2024) model of GM has two exchanging compartments: a collection of neurites modeled as randomly oriented infinitely long sticks, and the extracellular space modeled as an isotropic Gaussian medium. They are based on Kärger’s analytical framework of barrier-limited exchange between two Gaussian compartments (Kärger, 1985). They differ in that the NEXI signal equations were derived under the narrow pulse approximation, whereas SMEX explicitly accounts for the finite width of the diffusion gradients. Fitting NEXI/SMEX to diffusion MRI data yields estimates of neurite water fraction (*f*), intra- and extracellular diffusivities (*D_i_* and *D_e_*, respectively), and exchange time between the neurite and extracellular spaces (*t_ex_*), all of which are of potential clinical or biological relevance. In particular, the exchange time is assumed to relate to cell membrane permeability, and has been shown to correlate well (but not exclusively) with myelination in the cortex (Hertanu et al., 2025; Uhl et al., 2025).

NEXI has been successfully applied in murine brains *in vivo* (Jelescu et al., 2022) and *ex vivo* (Olesen et al., 2022), and human brains both *post mortem* (Hertanu et al., 2023) and *in vivo*, using Connectom (Chan et al., 2025; Uhl et al., 2024) and clinical scanners (Hertanu et al., 2025; Uhl et al., 2025). These studies demonstrate the feasibility of estimating human cortical microstructural parameters *in vivo*. More recently, NEXI has also been applied in disease contexts, namely Glucose Transporter-1 Deficiency Syndrome in mice (Hertanu et al., 2026) and hTyr rat model of Parkinson’s Disease (Pérot et al., 2026).

Despite NEXI’s demonstrated potential, the validity of its assumptions remains insufficiently characterized. Structural features commonly observed in brain tissue, such as neurite beading and undulation, may violate the Gaussian diffusion assumption of each compartment underlying the Kärger model (H.-H. Lee et al., 2020; Nguyen-Duc, Brammerloh, et al., 2026; Novikov et al., 2014), thereby affecting the accuracy of the estimated NEXI microstructural parameters. Additional cellular features, such as somas (Olesen et al., 2022) and dendritic spines (Chakwizira et al., 2026; Şimşek et al., 2025), can further influence the accuracy of NEXI-derived microstructural estimates. In particular, exchange between dendritic spines and the shaft have recently been shown to produce an MRI signal signature similar to that due to inter-compartmental exchange across the membrane, although ultra-strong diffusion gradients (preclinical-grade and higher) would be needed to observe this effect experimentally (Chakwizira et al., 2026; Şimşek et al., 2025).

Previous validation efforts for NEXI have not accounted for the effects of realistic neuronal geometries: some studies generated synthetic data and fitted it using the same NEXI analytical model (Chan et al., 2025; Jelescu et al., 2022; Uhl et al., 2024), while others employed numerical phantoms with realistic neuronal geometries but restricted the MRI signal to the intracellular compartment, thereby excluding exchange with the extracellular space (Olesen et al., 2022; Şimşek et al., 2025).

To address such limitations, numerical phantoms of packed cells into a synthetic tissue voxel have emerged as a powerful framework for validating biophysical models (Fieremans & Lee, 2018; Ginsburger et al., 2019; Nguyen-Duc, Brammerloh, et al., 2026). They allow the generation of tissue-like substrates with known ground-truth properties, in which the diffusion process can be simulated, typically with Monte Carlo methods. The resulting synthetic MRI signals can then be fitted with biophysical models, allowing a direct comparison between estimated parameters and their true ground-truth values (Jelescu et al., 2020; Nguyen-Duc, Uhl, et al., 2026). Importantly, numerical phantoms are particularly useful for studying permeability, as water exchange across the cellular membrane can be explicitly modeled and systematically varied (Gardier et al., 2023).

In this work, we use numerical phantoms to evaluate the performance of NEXI in substrates approximating key aspects of GM microstructure, including beading and undulating neurites, and spanning a range of membrane permeabilities. We additionally included somas, which are not accounted for in the NEXI model, and varied the level of neurite orientation dispersion to reflect different GM architectures. We did not include dendritic spines in our simulations, as previous work has shown that spine-related sensitivity arises primarily at short diffusion times (Şimşek et al., 2025), which are generally not accessible with the clinical or preclinical diffusion MRI sequences used for NEXI. In addition, reliable numerical simulators explicitly incorporating spines are currently not available.

## 2 Methods

### 2.1 Numerical substrate generation

Numerical substrates representing GM were generated within a ${\left(100\mu \text{m}\right)}^{3}$ voxel using the CATERPillar toolbox (Nguyen-Duc, Brammerloh, et al., 2026) (v. 05/08/2025). This generator models neurites as chains of overlapping spheres.

The generated substrates consisted of three orthogonal fiber populations to reproduce the high orientation dispersion expected in GM (Lampinen et al., 2020; Salo et al., 2021), with a target neurite volume fraction (*f*) of 65%. The neurite radius distribution was aimed to peak around 1μm , in agreement with reported values of 0.5–1μm in humans and rodents (Aird-Rossiter, Zhang, et al., 2026; Palombo et al., 2019). Since CATERPillar inflates neurites in a final step to achieve the target volume fraction, the substrate generation was initialized with a gamma distribution of radii using a shape parameter α=1.60 and a scale parameter β=0.30 (corresponding to a mode of approximately 0.18μm ) for all substrates.

To increase the realism of neurites, we incorporated two morphological features: beading (local radius fluctuations along the neurite) and undulation (deviation of the centerline from a straight path).

In CATERpillar, beading amplitude *A* is defined as a fraction of the neurite’s initial radius. In this work, neurite beading is quantified by the coefficient of variation of the radius $CV=\frac{\text{std}\left(r\right)}{\text{mean}\left(r\right)}$ (H. H. Lee et al., 2019; H.-H. Lee et al., 2024). As a representative baseline value, we set CATERPillar parameter *A* to 0.2 times the radius, which corresponds to a CV of ~0.2 . This choice is motivated by electron microscopy studies of axons (as quantitative data on neurite beading are scarce), which report beading amplitudes of 0.2–0.3 times the radius (CV~0.2 (H. H. Lee et al., 2019; H.-H. Lee et al., 2024)), with substantially larger beading amplitudes of up to ~0.57 times the radius observed only under pathological conditions such as ischemia (Budde & Frank, 2010).

In CATERPillar, undulation is controlled by parameter ε, which modulates the strength of attraction toward a given target (Nguyen-Duc, Brammerloh, et al., 2026). In this work, we use the term ‘undulation’ (und) to refer to the ratio of total process length divided by its end-to-end distance, rather than tortuosity—a commonly used term for processes undulation (Aird-Rossiter, Zhang, et al., 2026)—to avoid confusion with extracellular tortuosity which is defined as the ratio of free diffusivity to effective hindered diffusivity (e.g., *D_free_*/*D_e_*) (Jespersen et al., 2007; Kaden et al., 2016; H.-H. Lee et al., 2020; Novikov, 2021). As a representative baseline value, we set CATERPillar ε to 0.2, which yields an undulation factor of ~1.05 . This value is consistent with microscopy-reconstructed neurons, which report median branch undulation factors of ~1.30−1.35 for pyramidal and GABAergic neurons in humans (Aird-Rossiter, Zhang, et al., 2026), and 1.10–1.15 for dendrites and 1.10–1.20 for axons in rats (Stepanyants et al., 2004).

This substrate was considered the most realistic and used as the default (Table 1). It was then systematically modified by varying individual CATERPillar parameters: beading amplitude *A* from 0 to 0.6 times the initial radius (step 0.1), corresponding to CV = 0.18-0.37; undulation amplitude ε from 0 to 0.4 (step 0.1), corresponding to undulation factors 1.00-1.30 (Table 1). The level of neurite orientation dispersion is characterized by the *l* = 2 rotational invariant *p*_2_ and related to the dispersion angle Ψ by $p_{2}=\frac{3\langle {\text{cos}}^{2}\Psi \rangle -1}{2}$ (with *p*_2_ = 0 corresponding to a fully isotropic orientation distribution and *p*_2_ = 1 to perfectly aligned neurites). Orientation dispersion was modified by reducing the number of orthogonal fiber populations from three to two. Because neurite growth and packing is easier under certain morphological conditions, requiring less inflation in the final step to maintain a comparable radius distribution and volume fraction, we used shape and scale parameters of α=5.00 and β=0.20 for the radius distribution, with a neurite volume fraction of 40% for the non-undulated case and for the two-fiber case; and a target neurite volume fraction of 40% for the undulation case ε = 0.3 and ε = 0.4.

**Table 1.** Target CATERPillar substrate parameters, including beading (amplitude *A* and expected final CV), undulation (*ϵ* and expected final undulation ratio *und*), number of fiber populations, soma fraction *f_s_*, neurite fraction *f*, neurite membrane permeability κ (μm/s ), and extracellular space (ECS) tortuosity and gap distance (nm).

	Beading		Undulation						ECS	
Condition	*A*	CV	*ϵ*	und	# Fibers	*f_s_*	*f*	κ(μm/s)	Tortuosity	Gap distance
Default	0.2	≈ 0.24	0.2	≈ 1.05	3	0	0.38	20	1.16	370
Permeability increase	0.2	≈ 0.24	0.2	≈ 1.05	3	0	0.38	1–100	1.16	370
Beading increase	0–0.6	≈ 0.18–0.37	0.2	≈ 1.05	3	0	0.35–0.39	20	1.14–1.17	≈ 367
Undulation increase	0.2	≈ 0.24	0–0.4	≈ 1.00–1.30	3	0	0.31–0.42	20	1.10–1.18	≈ 363
Orientation dispersion decrease	0.2	≈ 0.24	0.2	≈ 1.05	2–3	0	0.38–0.40	20	1.16–1.18	≈ 368
With somas	0.2	≈ 0.24	0.2	≈ 1.05	3	0.05–0.1	0.30–0.35	20	1.15–1.16	≈ 371

For the default substrate, we evaluated several levels of membrane permeability: κ=10,20,30, and 50μm/s , in agreement with experimental membrane permeability values in healthy cells (Boss et al., 2013; Harkins et al., 2009; Stanisz et al., 1997). To further investigate the dynamic range and behavior of the model, we extended the explored permeability range by approximately one order of magnitude on both sides by additionally evaluating κ = 1 and 100μm/s . For the remaining substrates, κ was kept at 20μm/s .

Finally, we also generated two substrates that included somas (Table 1). These substrates shared the same features as the default configuration but incorporated a population of spherical somas uniformly distributed in the voxel. The soma volume fractions (*f_s_*) in these two substrates were 5% and 10%, respectively, and the soma radius distribution had mean 7.1μm and standard deviation 3.6 μm, consistent with reported values for pyramidal cells in rats (Aird-Rossiter, Zhang, et al., 2026; Olesen et al., 2022). In our substrates, somas were disconnected from neurites, as the former were placed using CATERPillar’s glial cell population generator and the latter using the axon population generator (as CATERPillar is primarily designed for WM substrates). Nonetheless, having disconnected somas also enables us to ensure comparability of this particular substrate with the others, as the main difference is indeed only the addition of spherical components. Furthermore, soma-dendrite exchange is typically quite slow (De Riedmatten et al., 2024). In these substrates, soma membranes were first assumed impermeable, as in SANDI (Palombo et al., 2020), and then also assigned a permeability of 20μm/s , the same as the neurites’.

A summary of the most important parameters used to generate the substrates is presented in Table 1. The tortuosity of the extracellular space was calculated as $\sqrt{D_{\text{free}}/D_{e}}$ (Jespersen et al., 2007; Nicholson, 2001), where *D_e_* is the effective hindered diffusivity obtained from the Monte Carlo simulations and $D_{\text{free}}$ is the free diffusivity input to the simulator. The extracellular gap distance was estimated by binarizing the substrate swc files and converting them to NIfTI images. A Euclidean distance was computed within the extracellular space to obtain the distance from each extracellular voxel to the nearest cellular boundary. The local gap distance was defined as twice this distance, and a representative gap distance was taken as the mode of the resulting distribution.

As a supplementary analysis, we further assessed the impact of structural disorder by comparing the default substrate, which exhibits low levels of beading and undulation, with substrates showing high levels of beading and undulation (CATERPillar beading amplitude A=0.6 , corresponding to a CV of 0.36, and CATERPillar undulation ε=0.4 , corresponding to und=1.27 ).

### 2.2 Monte Carlo simulations

To estimate MRI signals from the generated synthetic substrates, we performed Monte Carlo simulations of water diffusion. We used a simulator adapted to permeable cell membranes between intra- and extracellular spaces (Gardier et al., 2023; Rafael-Patino et al., 2020). The simulator was further modified to handle cellular structures defined by overlapping spheres instead of triangular meshes, as implemented for CATERPillar (Nguyen-Duc, Brammerloh, et al., 2026; Nguyen-Duc, Uhl, et al., 2026). Analyses were conducted to ensure that mass conservation was not violated in complex permeable substrates (Supplementary Fig. S1).

The trajectory simulations were performed for a total time of TE=52 ms and repeated three times. The intrinsic/free diffusivities set in the simulator in the intra- (*D_free_*_,_*_i_*) and extracellular (*D_free_*_,_*_e_*) spaces were $2\mu {\text{m}}^{2}\text{/ms}$ and $1\mu {\text{m}}^{2}\text{/ms}$ , respectively. This choice was made to compensate for the relatively low extracellular tortuosity of our numerical substrates compared with biological tissue, such that the resulting effective diffusivities at finite diffusion times remain consistent with the commonly reported condition *D_i_* > *D_e_* (Dhital et al., 2019; Jelescu et al., 2020; Kunz et al., 2018) and within the range of values reported in the literature (Jallais et al., 2026; Jelescu et al., 2022; Uhl et al., 2025). Each simulation was initialized with a walker density of 1 walker/μ*m*^3^, corresponding to 10^6^ walkers, distributed homogeneously inside and outside the neurites. This particle density was in line with previously reported benchmark values (Fieremans & Lee, 2018) and was further confirmed by the stability of the estimated metrics with increasing walker density (Supplementary Fig. S2). The step size of each walker in one direction δx was set to ~0.06μm , approximately one-tenth of the minimum neurite radius on the final substrates and smaller than the average gap size of the extra-cellular space (Table 1). The corresponding simulation step duration Δ*t* was =1μs , computed from $\delta x=\sqrt{2D_{free,i}\Delta t},$ leading to a total number of steps *T* = 64800 (as Δt=TE/T ).

Synthetic diffusion-weighted signals were generated from the resulting trajectory simulations assuming a pulsed-gradient spin-echo (PGSE) sequence and a feasible preclinical $(G_{max}\;\sim 600\text{mT/m}$ ) *in vivo* protocol adapted from previous works (Oliveira et al., 2026; Pérot et al., 2026), with *b*-values of 1 (12 directions), 2 (16 directions), 3.5 (24 directions), 5 (30 directions), and $7\text{ ms/}\mu {\text{m}}^{2}$ (40 directions). The diffusion times (Δ) were 15, 26, and 38 ms, with a gradient duration (δ) of 4.5 ms to be within the narrow pulse approximation.

We further simulated a clinically feasible protocol, following Uhl et al. (2025) (characterized by limited gradient performance, which constrains the achievable b-values and requires longer δ and Δ). Specifically, diffusion times (Δ) of 28.3, 36, 45, 55, and 65 ms were simulated. For Δ = 28.3 ms and 36 ms, *b*-values of 1 and 2 $\text{ms/}\mu {\text{m}}^{2}$ were used; for Δ = 45 ms, *b*-values of 1, 2, 3.2, and 4.44 $\text{ms/}\mu {\text{m}}^{2}$; and for Δ = 55 ms and 65 ms, *b*-values of 1, 2, 3.2, and 5 $\text{ms/}\mu {\text{m}}^{2}$. Each Δ included 20 gradient directions per shell, with δ = 16.5 ms. In the Monte Carlo simulations for the clinical protocol, the number of steps *T* was increased such that the total simulation duration corresponded to the longest diffusion time (Δ = 65 ms) plus δ=16.5 ms, i.e., 81.5 ms.

### 2.3 Analysis

The NEXI model was fitted to the magnitude of the generated complex-valued diffusion-weighted signals using the implementation available in the Swiss Knife Toolbox (https://github.com/QuentinUhl/graymatter_swissknife (Jelescu et al., 2022; Uhl et al., 2024, 2025)). NEXI estimates for *f*, *D_i_*, *D_e_* and *t_ex_* were compared to ground-truth values of the substrate. Ground-truth values for *f* were directly obtained from the geometric properties of the neurites in the substrate. The ground-truth effective intracellular and extracellular diffusivities were obtained from the simulated walker trajectories for each substrate without permeability, computed at finite diffusion times, and averaged across diffusion times. For *t_ex_*, ground truth values were derived from the relationship between membrane permeability and neurite surface-to-volume ratio (*S*/*V*), given by $\tau_{i}=\frac{1}{\kappa }\frac{V}{S}$, where τ*_i_* is the intracellular residence time, and related to the NEXI exchange time through $t_{ex}\;=\left(1-f\right)\tau_{i}$ (Fieremans & Lee, 2018; Fieremans et al., 2010; H.-H. Lee et al., 2025). This ground-truth estimate was itself further validated against exchange times computed directly from the simulated intracellular and extracellular residence times of particle trajectories, yielding differences of 1 ms for κ = 20 or 30μm/s and of 10 ms for κ=10μm/s (Supplementary Fig. S1). This discrepancy at low permeability may reflect a limited sensitivity of our protocol to large exchange times, given the maximum diffusion time of 38 ms; and to the fact that at low permeability there are fewer membrane-crossings, which increases the variability of the trajectory-based estimate and may require a larger number of walkers to obtain a more robust ground-truth value. Based on the close agreement at κ=20μm/s , which is the default value for all substrates except the ones where we vary permeability, we chose to report the ground-truth estimated for *t_ex_* computed from the membrane permeability and neurite surface-to-volume ratio.

To further characterize the microstructure underlying the simulated voxel, we assessed the time dependence of mean diffusivity (MD) and mean kurtosis (MK). To this end, diffusion kurtosis imaging (DKI) was performed independently at each diffusion time using the TMI toolbox (Veraart et al., 2013).

For comparison with the full NEXI model estimate, exchange time *t_ex_* was also computed from the MK time dependence (Fieremans et al., 2010; Jensen & Helpern, 2010):

$$K\left(\Delta \right)=K_{0}\frac{2t_{ex}}{\Delta }\;\left[1-\frac{t_{ex}}{\Delta }\left(1-e^{-\Delta /t_{ex}}\right)\right]$$ (1)

To evaluate estimation errors under realistic noise conditions, noise was included in signals generated at different permeability levels in the default substrate. Noise was not included for all substrate configurations, as the impact of noise on NEXI estimates has already been characterized in prior work (Jelescu et al., 2022; Uhl et al., 2024) and is beyond the scope of this study; therefore, the default configuration was used as a representative case. We added Rician noise (SNR of 50), by introducing Gaussian noise to the real and imaginary parts of the noise-free signals and then taking the magnitude. The procedure was repeated 500 times, and the data from each realization were fitted as described above. In these cases, in the NEXI fit, the Rician noise floor was accounted for (Uhl et al., 2024).

## 3 Results

### 3.1 Numerical substrates generation

The generated substrates broadly matched the targeted morphological parameters (Fig. 1), with a few expected deviations. Final neurite radii were slightly larger than the target distribution due to the inflation step applied in CATERPillar to reach the desired volume fraction. By undershooting the target distribution, we obtained a final mean radius within the reported range of 0.5–1μm in humans and rodents (Aird-Rossiter, Zhang, et al., 2026; Palombo et al., 2019). The target neurite volume fraction of 65%, however, was not achieved, with the final value ranging 31 - 42%, depending on the substrate (Fig. 1). This limitation arises from the difficulty of densely packing neurites (a challenge commonly encountered in GM substrate generation (Fieremans & Lee, 2018; Ginsburger et al., 2019)), particularly when incorporating three different fiber orientations, as in our case. Nevertheless, the achieved neurite volume fractions across substrates are comparable to literature neurite density estimates *in vivo* using either NEXI or SANDI (Jelescu et al., 2022; Schiavi et al., 2023; Uhl et al., 2025).

**Fig. 1.**
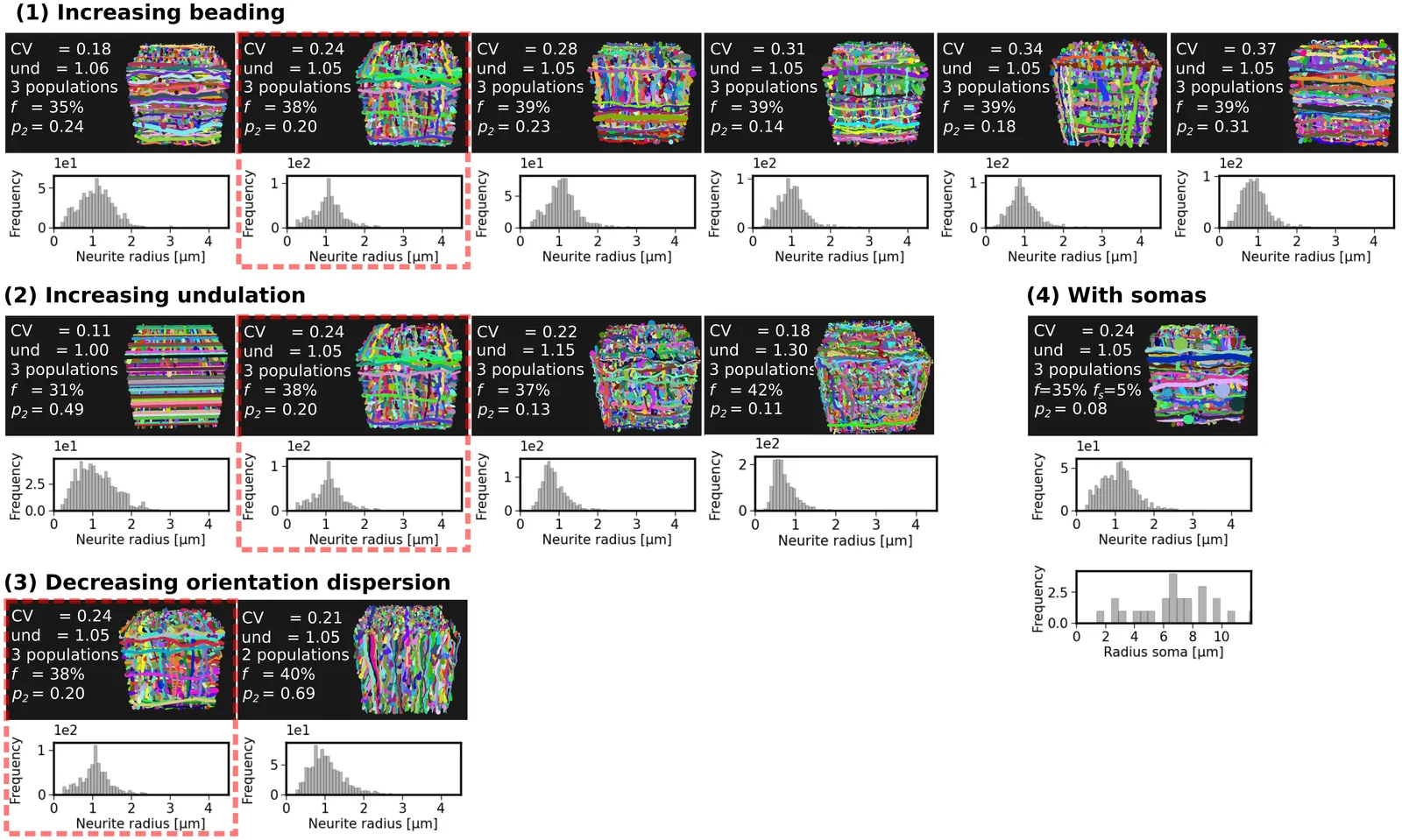
Generated substrates. Substrates mimicking GM were generated with different morphological features: (1) increasing beading level, (2) increasing undulation level, (3) decreasing orientation dispersion, and (4) presence of somas. The default substrate is highlighted in a red box with dashed lines. Beading level was defined as the coefficient of variation of the radius, given by $CV=\frac{\text{std}\left(r\right)}{\text{mean}\left(r\right)}$ (H. H. Lee et al., 2019), and undulation was defined as the ratio between total neurite length and its end-to-end distance; both averaged across neurites. The resulting neurite volume fraction (*f*), soma volume fraction (*f_s_*), orientation dispersion (*p*_2_), the distribution of mean radius along each neurite, and distribution of soma radius are shown for each case.

The different substrates were broadly comparable, particularly in terms of the neurite radius distribution. Orientation dispersion, quantified by *p*_2_, fell within the expected range for GM (~ 0.13–0.30) (Lampinen et al., 2020; Salo et al., 2021) for most substrates. Removing undulation increased *p*_2_, as neurites became straighter and exhibited less local directional variability. The neurite volume fraction *f* increased slightly with stronger beading (reflecting the larger neurite volume) and with larger undulation (as wavier neurites take up more space and can grow around obstacles, facilitating denser packing). Note that even when beading is not introduced (*A* = 0), neurites still undergo local constrictions to pass through narrow gaps, leading to a final CV different from 0 (= 0.18).

### 3.2 Impact of cell membrane permeability

First, we evaluated NEXI performance on the default substrate (beading CV = 0.24, undulation = 1.05) across varying membrane permeabilities.

For permeabilities of κ=10,20,30 , and 50μm/s , the estimated *t_ex_* ranged from 9 to 49 ms, following the expected trend from theoretical predictions (Fig. 2a), with biases of +26%, +15%, +14%, and +20% compared to the ground-truth computed from the substrate, respectively. For non-zero but very low permeability κ=1μm/s , the estimated *t_ex_* saturates near the maximum value allowed in the fitting procedure, as this regime is already close to impermeable. For very large permeability (κ=100μm/s ), *t_ex_* could still be recovered with a bias of +27%.

**Fig. 2.**
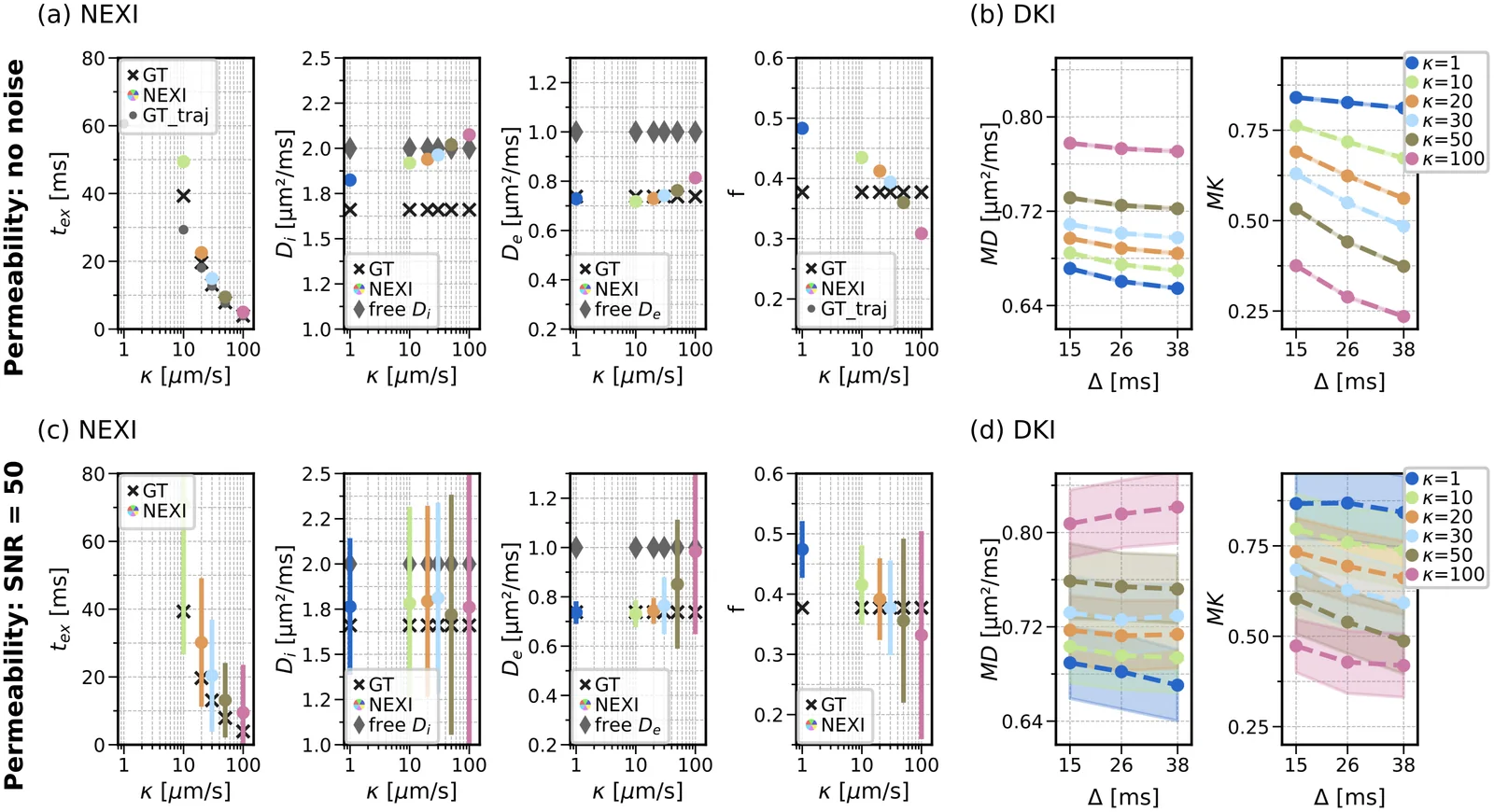
NEXI performance across permeability levels. NEXI parameter *D_e_* agreed with trajectory-derived values (black markers) and *D_i_* was more overestimated with larger permeability. NEXI parameters *D_e_* and *D_i_* were lower than the intrinsic diffusivity (gray markers), reflecting hindered diffusion due to neurite irregularities (a). *t_ex_* followed theoretical predictions, and *f* was most accurate for intermediate permeability values (a). MD showed weak time dependence, while MK exhibited a pronounced time dependence that increased with permeability (b). With added noise, the same overall trends were preserved, but variability increased in both NEXI (c) and DKI (d) estimates. Black cross markers indicate ground-truth (GT) values (for *t_ex_* we show the GT computed from the substrate geometry using the relationship between membrane permeability and neurite surface-to-volume ratio (black crosses) and computed from the particle trajectories (gray circles)); and gray diamond markers indicate *D_i_* and *D_e_*, the intrinsic/free diffusivities specified in the simulator. The error bars represent the standard deviation of the estimates obtained from 500 noise realizations added to the noiseless Monte Carlo signal. GT and estimated exchange times are not shown for κ=1μm/s , as this is a nearly impermeable case, leading to exchange times well beyond the range of interest.

NEXI estimates of *D_i_* and *D_e_* were lower than the intrinsic diffusivities specified in the simulator (gray markers) (Fig. 2a), as expected due to neurite irregularities and orientation dispersion, which introduce additional obstacles in both intra- and extracellular compartments and thereby lead to more hindered diffusion. *D_e_* closely matched the diffusivities computed directly from the simulated extracellular walker trajectories (black markers) and remained stable across permeability levels (bias ~1%), although the overestimation increased to 10% at κ=100μm/s . In contrast, *D_i_* was consistently overestimated, with the bias increasing from approximately 10% at low permeability to 25% at (κ=100μm/s ), as the estimated values approached the intrinsic/free diffusivity with increasing membrane permeability. The estimation of *f* was most accurate for intermediate permeability values. Lower permeability values led to an overestimation of *f*, whereas higher permeability values resulted in an underestimation.

Consistent with the NEXI parameters, the diffusion kurtosis metrics also reflected the effect of exchange (Fig. 2b): MK exhibited a pronounced time dependence that increased with increasing permeability, whereas MD showed only a weak time dependence with slopes largely preserved across permeability levels (Table 2a). This behavior is in line with the expected dominant influence of exchange over structural disorder (Fieremans et al., 2010; Jelescu et al., 2022). Exchange times derived from MK time dependence were on average about 8 ms longer than the corresponding NEXI estimates (Table 3a).

**Table 2.** Slopes of MD (in $10^{-3}\mu {\text{m}}^{2}{\text{/ms}}^{2}$) and MK (10^−3^/ms) time dependence for different permeability levels.

(a) Noise free (see Fig. 2b)		
	Slope MD	Slope MK
κ=1μm/s	-0.73	-1.27
κ=10μm/s	-0.65	-3.89
κ=20μm/s	-0.56	-5.59
κ=30μm/s	-0.49	-6.30
κ=50μm/s	-0.40	-6.88
κ=100μm/s	-0.30	-6.08
(b) With noise (SNR = 50) (see Fig. 2d)		
	Slope MD	Slope MK
κ=1μm/s	-0.87 ± 0.17*	-1.32 ± 0.57*
κ=10μm/s	-0.67 ± 0.17*	-3.21 ± 0.68*
κ=20μm/s	-0.19 ± 0.22	-3.48 ± 0.88*
κ=30μm/s	0.54 ± 0.30	-1.36 ± 1.07
κ=50μm/s	-0.44 ± 0.32	-5.81 ± 0.91*
κ=100μm/s	0.74 ± 0.62	-2.07 ± 1.00

For the case with noise, values are reported as mean ± standard error of the mean across noise realizations. Statistical significance was assessed using a one-sample t-test against zero; slopes significantly different from zero (p < 0.05) are marked with (*).

**Table 3.** Exchange time *t_ex_* (ms): ground-truth (GT), NEXI-estimated, and MK time-dependence–derived values for different permeability levels.

(a) Noise free			
	GT *t_ex_*	NEXI ${\hat{t}}_{ex}$	MK ${\hat{t}}_{ex}$
κ=1μm/s	393	150	150
κ=10μm/s	39	49	57
κ=20μm/s	20	23	32
κ=30μm/s	13	15	24
κ=50μm/s	8	9	16
κ=100μm/s	4	5	11
(b) With noise (SNR = 50)			
	GT *t_ex_*	NEXI ${\hat{t}}_{ex}$	MK ${\hat{t}}_{ex}$
κ=1μm/s	393	140 ± 26	105 ± 5
κ=10μm/s	39	71 ± 45	92 ± 6
κ=20μm/s	20	30 ± 19	75 ± 8
κ=30μm/s	13	20 ± 16	98 ± 10
κ=50μm/s	8	13 ± 11	47 ± 11
κ=100μm/s	4	10 ± 14	97 ± 29

For the case with noise, values are reported as mean ± standard error of the mean across noise realizations.

In the case of finite SNR (SNR = 50), NEXI estimates of *t_ex_* showed bias of +81%, +53%, +56%, and +67% for permeabilities of κ=10,20,30 , and 50μm/s , respectively (Fig. 2c), highlighting the important impact of noise on parameter estimation. Among the four parameters, *D_i_* and *t_ex_* showed the largest variability, as expected given their higher estimation uncertainty (Jallais et al., 2026; Uhl et al., 2024). Nevertheless, the ground-truth parameter values still fall within the corresponding uncertainty ranges of the estimates. Similarly, MD and MK also exhibited increased uncertainty under noisy conditions (Fig. 2d), but the expected trend of stronger MK time dependence with higher permeability was still observed (Table 2b). As expected, with a lower SNR (=20), both the variability and the bias of the NEXI estimates increased further (Supplementary Fig. S3c). The time dependence of MD and MK becomes unstable and departs from the expected physical behavior (Supplementary Fig. S3d). The exchange times estimated from MK time dependence deviated more from the ground-truth than the NEXI estimates (Table 3b).

### 3.3 Impact of neurite irregularities

We next evaluated the impact of neurite beading and undulation on NEXI estimates, using a reference permeability of κ=20μm/s (Fig. 3).

**Fig. 3.**
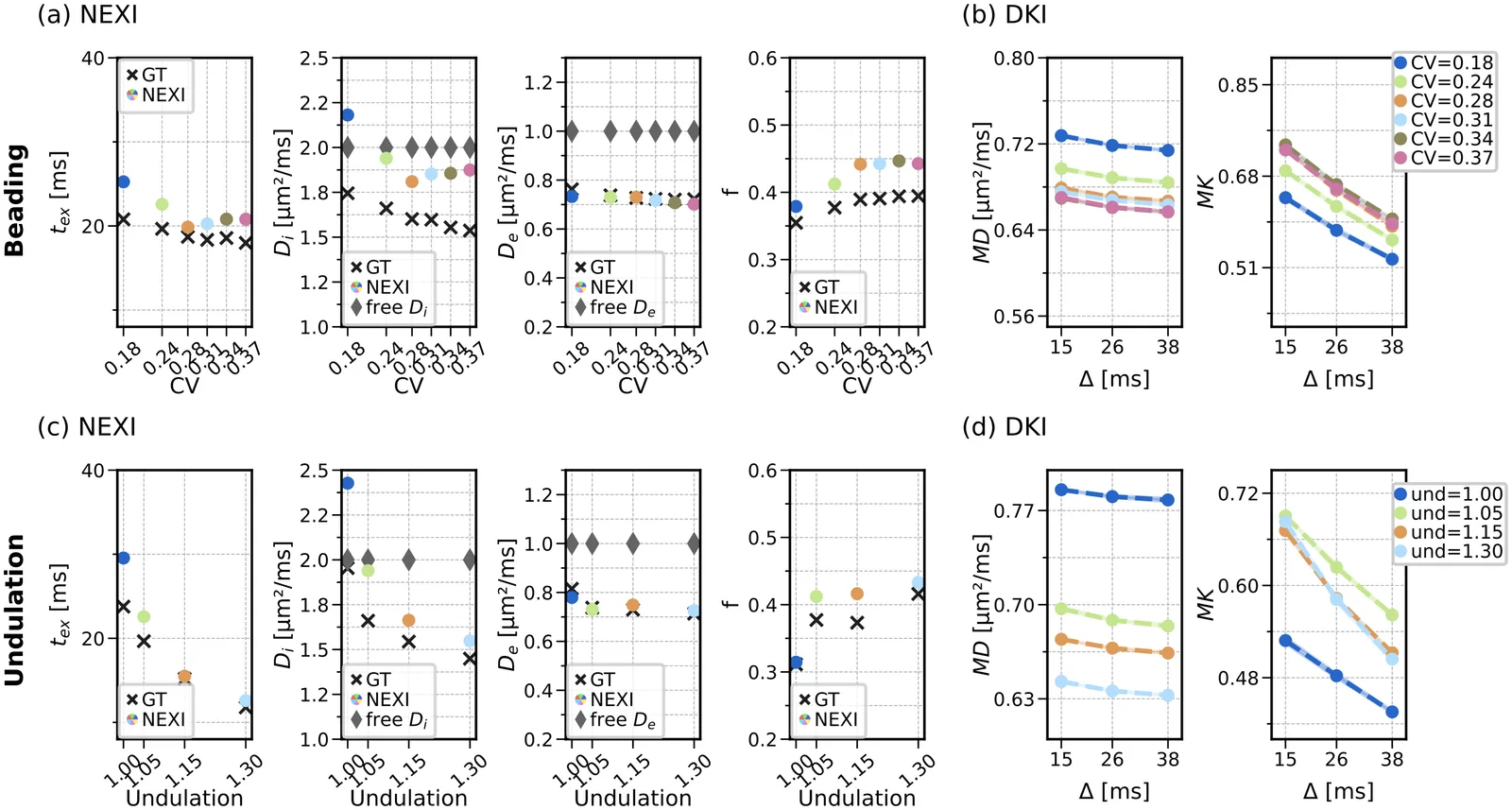
NEXI performance with neurite irregularities. NEXI parameter *f* showed limited sensitivity to neurite beading, showing only a small increase in bias (from +7% to +12%), while *t_ex_* remained relatively stable (approximately +13%), as beading level (CV) rose from 0.18 to 0.37 (a). NEXI parameter *D_e_* remained constant and in agreement with the ground-truth. The ground-truth *D_i_* in general decreased with increasing beading, consistent with restricted intracellular diffusion, but the estimated *D_i_* decreased and then slightly increased for large beading levels and therefore did not capture this dependence (a). The restricted intracellular diffusion was similarly reflected in a decrease of MD and an increase of MK (b). The ground-truth *t_ex_* were much more sensitive to undulation (c) than to beading, while NEXI estimates successfully estimated these variations. Remarkably, the largest bias was found for *t_ex_* and *D_i_* estimates at the lowest undulation level (c). Undulation affected diffusion kurtosis metrics similarly to neurite beading (d). Black cross markers indicate ground-truth (GT) values and gray diamond markers indicate *D_i_* and *D_e_*, the intrinsic/free diffusivities specified in the simulator. Beading level was defined as the coefficient of variation of the radius, given by $CV=\frac{\text{std}\left(r\right)}{\text{mean}\left(r\right)}$ (H. H. Lee et al., 2019), and undulation was defined as the ratio between the total neurite length and its end-to-end distance.

NEXI estimates *t_ex_* and *f* showed limited sensitivity to beading, and although some bias was present, it increased only marginally with stronger beading from +7% to +12% for *f*, while remaining relatively stable for *t_ex_* (approximately +13%), as beading (CV) increased from 0.18 to 0.37.

NEXI estimate *D_e_*, which was consistently lower than the intrinsic diffusivity due to neurite packing, as noted previously, agreed with the ground-truth. Also, it remained relatively stable across all beading levels, with a slight decrease as beading increased, likely due to increased hindrance in the extracellular space. Ground-truth *D_i_* systematically decreased as beading increased, consistent with reduced water mobility in the neurites caused by larger beads (Budde & Frank, 2010; H.-H. Lee et al., 2024; Nguyen-Duc, Brammerloh, et al., 2026). However, NEXI estimate *D_i_* did not capture this trend, instead producing increasingly overestimated values as beading increased, a behavior similar to that observed for *t_ex_*.

Consistent with these NEXI estimates trends, diffusion kurtosis metrics showed a decrease in MD and an increase in MK with increasing beading, reflecting the increased hindrance and non-Gaussian diffusion caused by the irregularities (Fig. 3b). The time dependence of both MD and MK remained consistent across all beading levels (Table 4a). Consequently, the corresponding exchange times were also similar across beading levels and were, on average, approximately 9 ms larger than the corresponding NEXI estimates (Table 5a).

**Table 4.** Slopes of MD (in $10^{-3}\mu {\text{m}}^{2}{\text{/ms}}^{2}$) and MK (10^−3^/ms) for different neurite irregularities.

(a) Beading (see Fig. 3b)		
	Slope MD	Slope MK
CV = 0.18	-0.59	-4.97
CV = 0.24	-0.56	-5.59
CV = 0.28	-0.53	-6.18
CV = 0.31	-0.52	-5.87
CV = 0.34	-0.56	-5.97
CV = 0.37	-0.57	-5.95
(b) Undulation (see Fig. 3d)		
	Slope MD	Slope MK
und = 1.00	-0.33	-4.03
und = 1.05	-0.56	-5.59
und = 1.15	-0.44	-6.89
und = 1.30	-0.45	-7.75

**Table 5.** Exchange time *t_ex_* (ms): ground-truth (GT), NEXI-estimated, and MK time-dependence–derived values for different neurite irregularities.

(a) Beading			
	GT *t_ex_*	NEXI ${\hat{t}}_{ex}$	MK ${\hat{t}}_{ex}$
CV = 0.18	21	25	34
CV = 0.24	20	23	32
CV = 0.28	19	20	30
CV = 0.31	18	20	33
CV = 0.34	19	21	32
CV = 0.37	18	21	32
(b) Undulation			
	GT *t_ex_*	NEXI ${\hat{t}}_{ex}$	MK ${\hat{t}}_{ex}$
und = 1.00	24	30	35
und = 1.05	20	23	32
und = 1.15	15	15	23
und = 1.30	12	13	20

Unlike beading, increasing undulation slightly increased the accuracy of *t_ex_* (with bias decreasing from +24% to +7% as the undulation increased from 1.00 to 1.30). The estimates of *f* showed no systematic trend with increasing undulation.

As in the case of beading, undulation increased hindrance to water diffusion within neurites, causing ground-truth *D_i_* to decrease as undulation increased (Fig. 3c). NEXI estimates of *D_i_* captured this trend, although *D_i_* was substantially overestimated at low undulation levels and became more accurate as undulation increased. Similar to beading, ground-truth *D_e_* was relatively stable across all undulation levels; and was accurately captured by the NEXI estimates of *D_e_*.

It is worth noting that in the cases of minimal beading and no undulation, *D_i_* estimates exceeded the intrinsic/free diffusivity. This is likely due to the lower orientation dispersion in these two substrates, which is expected to introduce larger errors in diffusivity estimation (Pizzolato et al., 2023).

Undulation affected diffusion kurtosis metrics similarly to neurite beading (Fig. 3d): MD decreased, and MK increased with greater undulation, consistent with hindered diffusion within irregular neurites. Unlike beading, however, the time dependence of MD and MK was more pronounced with stronger undulation (Table 4b). The exchange times estimated from MK time dependence ranged from 20 ms at the highest level of undulation to 35 ms at the lowest undulation (Table 5b); showing larger bias than the NEXI exchange-time estimates.

As a supplementary analysis, we further assessed the impact of pronounced structural disorder in a substrate with strong beading and undulation (Supplementary Fig. S5). Exchange time was more overestimated in the latter substrate compared to the default substrate. In fact, the bias increased from +50% to +79% when moving from κ = 20 to κ=10μm/s in the highly disordered substrate, compared with an increase from +15% to +26% in the least disordered substrate.

### 3.4 Impact of orientation dispersion and somas

NEXI assumes that neurites are randomly oriented; however, GM can exhibit varying levels of orientation dispersion (*p*_2_~ 0.13–0.30) (Lampinen et al., 2020; Salo et al., 2021). Thus, we evaluated NEXI in a substrate with reduced orientation dispersion.

The *t_ex_* estimate was less accurate in the substrate with lower orientation dispersion (higher *p*_2_) (+26%) compared to the more dispersed substrate (lower *p*_2_) (+16%) (Fig. 4a). On the contrary, the bias in *f* was lower in the substrate with lower orientation dispersion (6%) compared to the more dispersed substrate (9%). The ground-truth *D_i_* was higher in the substrate with lower orientation dispersion, and the NEXI estimates of *D_i_* followed the same trend, although with a larger bias in the less dispersed substrate. In contrast, the ground-truth *D_e_* was similar across the two evaluated substrates (Fig. 4a).

**Fig. 4.**
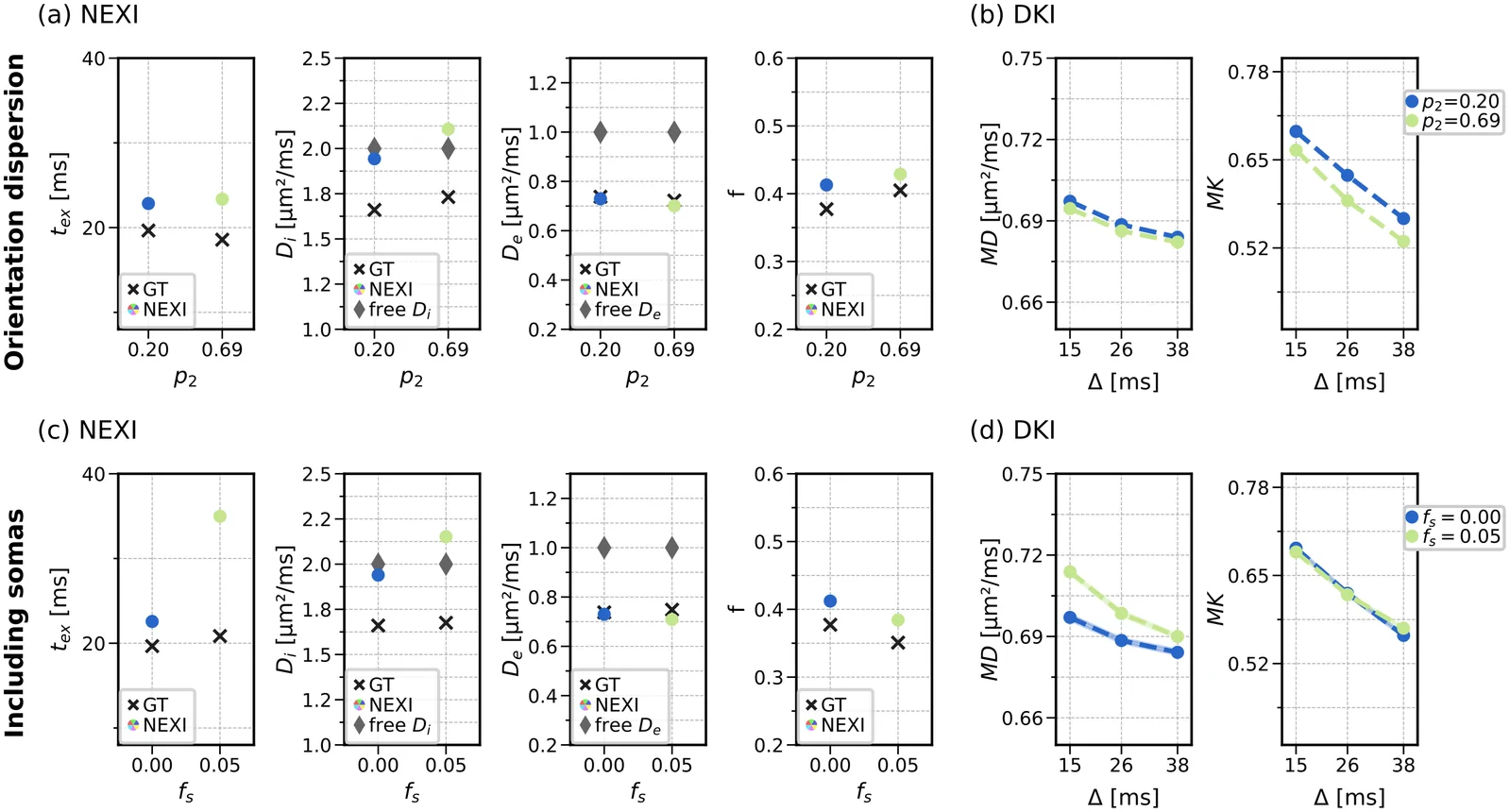
NEXI performance with different orientation dispersions and presence of somas. NEXI estimates of *t_ex_* and *D_i_* became less accurate in the substrate with lower orientation dispersion (a). On the contrary, the bias in *f* was lower in the substrate with lower orientation dispersion, and *D_e_* was accurately estimated in both cases (a). MD was similar across the two substrates, while MK was slightly lower in the substrate with lower orientation dispersion (b). The introduction of 5% somas, which are unaccounted for in NEXI, led to +68% bias in *t_ex_* and an overestimated *D_i_*, while *f* and *D_e_* were largely unaffected (c). MD increased due to the additional, less restricted diffusion within the somas, which was also reflected in a stronger MD time dependence. MK time dependence was unaffected (d). Black cross markers indicate ground-truth (GT) values and gray diamond markers indicate *D_i_* and *D_e_*, the intrinsic/free diffusivities specified in the simulator.

Regarding the diffusion kurtosis metrics, the higher *p*_2_ substrate resulted in similar MD and slightly lower MK (Fig. 4b). The time dependence of MK was more pronounced at low orientation dispersion (Table 6a), resulting in shorter exchange time estimates (Table 7a).

**Table 6.** Slopes of MD (in $10^{-3}\mu {\text{m}}^{2}{\text{/ms}}^{2}$) and MK (10^−3^/ms) for different orientation dispersions and presence of somas.

(a) Orientation dispersion (see Fig. 4b)		
	Slope MD	Slope MK
*p*_2_ = 0.20	-0.56	-5.59
*p*_2_ = 0.69	-0.48	-6.86
(b) Presence of somas (see Fig. 4d)		
	Slope MD	Slope MK
*f_s_* = 0.00	-0.56	-5.59
*f_s_* = 0.05	-1.03	-4.87

**Table 7.** Exchange time *t_ex_* (ms): ground-truth (GT), NEXI-estimated, and MK time-dependence–derived values for different orientation dispersions and presence of somas.

(a) Orientation dispersion			
	GT *t_ex_*	NEXI ${\hat{t}}_{ex}$	MK ${\hat{t}}_{ex}$
*p*_2_ = 0.20	20	23	32
*p*_2_ = 0.69	19	23	26
(b) Presence of somas			
	GT *t_ex_*	NEXI ${\hat{t}}_{ex}$	MK ${\hat{t}}_{ex}$
*f_s_* = 0.00	20	23	32
*f_s_* = 0.05	21	35	38

Next, we evaluated the case of impermeable somas at a soma volume fraction of 10%, for which the signal was dominated by the somas. The synthetic diffusion MRI signal reflects a restriction-dominated regime with higher signal at longer diffusion times (Supplementary Fig. S4a), which no longer reproduces the *in vivo* observation of an exchange-dominated decrease in signal with increasing diffusion time in GM (Jelescu et al., 2022; Olesen et al., 2022). As a result, the NEXI fit failed entirely. Introducing permeability in the soma membrane, with the same value as that of neurites, partially restored the expected signal time dependence, yielding a trend that was more consistent with experimental observations (Supplementary Fig. S4a). In this case, an exchange time between intra- and extra-cellular spaces could be estimated, unlike when somas were impermeable. Nevertheless, the estimated exchange time *t_ex_* showed high standard deviation across repeats and low accuracy relative to the ground-truth (Supplementary Fig. S4b). Indeed, a soma volume fraction of 10% in a substrate with neurite volume fraction of 30% represents an unrealistically high soma-to-neurite ratio of ~ 30%.

We therefore focused on the more realistic case of a soma volume fraction of 5% with soma–extracellular permeability κ=20μm/s . Combined with a neurite volume fraction of 35%, this yields a soma-to-neurite ratio of 14%, which is more consistent with histological reports showing soma ~9.4% , neurites ~66%, and a corresponding soma-to-neurite ratio of ~14% (Shapson-Coe et al., 2024). In this case, NEXI overestimated *t_ex_* by +68% (Fig. 4c), corresponding to a substantially larger bias compared to that observed in the absence of somas (+15%), but comparable to those arising from realistic finite SNR conditions. The introduction of somas led to an increase in *D_i_*. In contrast, the estimates of *f* and *D_e_* remained largely unaffected by the presence of somas (Fig. 4c).

Diffusion kurtosis metrics reflected the expected soma effects (Fig. 4d): the substrate containing 5% somas showed larger MD and exhibited increased MD time dependence due to highly restricted, non-Gaussian diffusion in the somas, as compared with the substrate without somas (Table 6b). MK exhibited the same exchange-dominated time dependence as in the case without somas, resulting in comparable MK-derived exchange time estimates (Table 7b). In particular, MK-derived exchange time was higher when somas were present than the case without somas, similar to NEXI estimates.

### 3.5 Performance for clinical acquisition protocols

To reflect a realistic clinical acquisition, we simulated a protocol suitable for clinical use (Uhl et al., 2025).

With the clinical protocol (Fig. 5a), the estimates of *t_ex_* were generally similar to those obtained with the preclinical protocol (Fig. 2a), with biases of -10%, -7%, +4% and +6% for κ=10,20,30, and 50μm/s , respectively. However, the estimates of *D_i_* and *D_e_* were more strongly affected, showing larger biases at higher permeability values (κ > 50 μm/s), reaching −14% and 19%, respectively, compared with −2% and 4% at lower permeabilities. The neurite fraction *f* was overestimated at very low permeability (κ≤ =10 10) and underestimated at very high permeability values (κ=100μm/s ), in the same way as with the preclinical protocol.

**Fig. 5.**
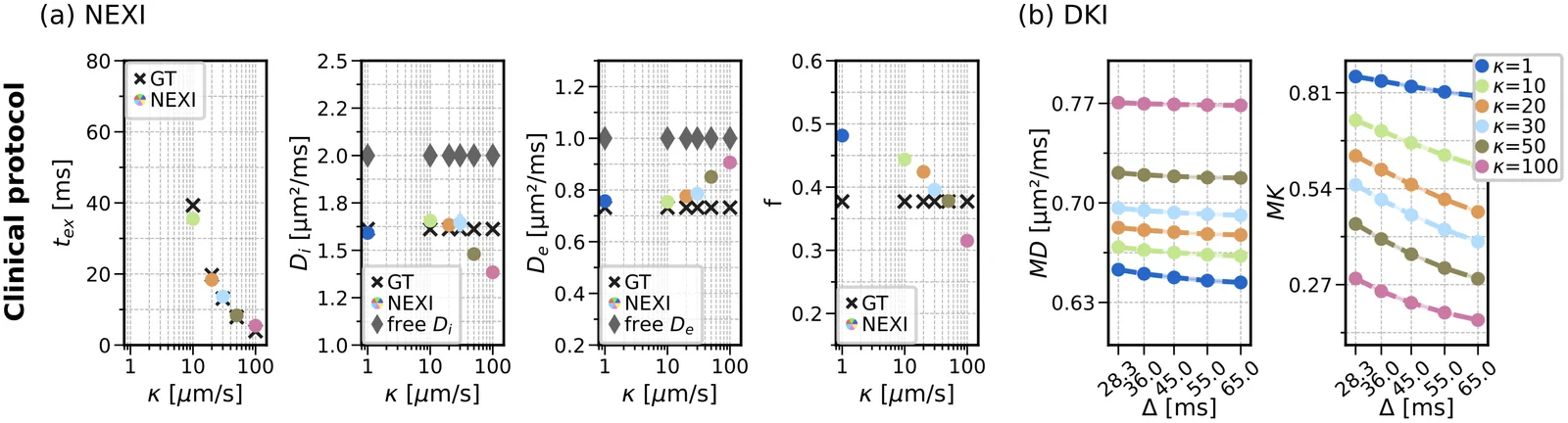
NEXI performance with a clinical protocol. With the clinical protocol (a), the estimated *t_ex_* values were generally similar to those obtained with the preclinical protocol (Fig. 2a). In contrast, the estimates of *D_i_*, *D_e_*, and *f* exhibited larger biases, particularly at higher permeability values. The estimate of *f* was also substantially biased at the lowest permeability. The diffusion kurtosis metrics also reflected the effect of exchange: MK showed a clear time dependence with increasing permeability, whereas MD time-dependence was much weaker (b). Black cross markers indicate ground-truth (GT) values and gray diamond markers indicate *D_i_* and *D_e_*, the intrinsic/free diffusivities specified in the simulator.

Similarly to the preclinical protocol, the diffusion kurtosis metrics also reflected the effect of exchange: MK showed a clear time dependence, whereas MD time-dependence was much weaker (Fig. 5b, Table 8). The exchange time estimated from MK time dependence deviated more from the ground-truth than the estimate obtained with NEXI (Table 9).

**Table 8.** Slopes of MD (in $10^{-3}\mu {\text{m}}^{2}{\text{/ms}}^{2}$) and MK (10^−3^/ms) for the clinical protocol.

	Slope MD	Slope MK
κ=1μm/s	-0.24	-1.53
κ=10μm/s	-0.17	-3.53
κ=20μm/s	-0.14	-4.30
κ=30μm/s	-0.14	-4.36
κ=50μm/s	-0.10	-4.20
κ=100μm/s	-0.05	-3.16

**Table 9.** Exchange time *t_ex_* (ms): ground-truth (GT), NEXI-estimated, and MK time-dependence–derived values for the clinical protocol.

	GT *t_ex_*	NEXI ${\hat{t}}_{ex}$	MK ${\hat{t}}_{ex}$
κ=1μm/s	393	150	150
κ=10μm/s	39	35	54
κ=20μm/s	20	18	34
κ=30μm/s	13	14	27
κ=50μm/s	8	8	19
κ=100μm/s	4	5	13

## 4 Discussion

In this work, we evaluated the performance of NEXI in numerical substrates incorporating varying levels of neurite beading and undulation, two levels of orientation dispersions, and a substrate with the inclusion of somas, with each factor varied separately, and across different membrane permeability levels.

We observe that NEXI exchange time estimates mirror the different permeability levels well and parameter estimates broadly follow changes in ground-truth parameters due to neurite irregularities and altered orientation dispersion. Non-negligible bias was nevertheless introduced by specific microstructural features: the largest bias in *t_ex_* was observed in the presence of somas, whereas *D_i_* was most affected by permeability, somas, and neurite beading. Despite these effects, NEXI estimates remained generally robust to the structural irregularities investigated. Notably, the estimation biases introduced by these microstructural features were often comparable or smaller than those arising from realistic finite SNR conditions.

Previous studies have demonstrated the potential of NEXI for *in vivo* applications in humans and rodents (Chan et al., 2025; Jelescu et al., 2022; Mezzano et al., 2025; Uhl et al., 2024, 2025), as well as animal models of disease (Hertanu et al., 2026; Pérot et al., 2026). The present work complements this body of evidence by addressing the important challenge of validating the model with numerical substrates that approximate key features of GM microstructure, evaluating how its assumptions and parameter estimates behave under more complex microstructural conditions, which can serve the interpretation of experimental results in human and rodent cohorts.

### 4.1 Substrate generation

To conduct this validation, we generated numerical substrates with the CATERPillar toolbox (Nguyen-Duc, Brammerloh, et al., 2026). One of the geometric features of GM that we aimed to reproduce was high orientation dispersion, though this proved challenging with CATERPillar, as this toolbox was primarily designed for WM. Therefore, rather than explicitly modeling orientation dispersion arising from neurites radiating from a soma, we approximated different levels of dispersion by combining two or three fiber populations with different orientations; however, intermediate dispersion levels could not be generated, limiting a full assessment of orientation dispersion impact on NEXI estimates.

Since somas are not accounted for in NEXI, we examined their potential influence on parameter estimates by including soma compartments in our simulated substrates. The soma-to-neurite ratio in our simulations (14%) was consistent with histological findings (~14%) (Shapson-Coe et al., 2024). However, the absolute soma and neurite volume fractions we were able to achieve (5% and 35%, respectively) remained at the lower end of reported values (somas: 5–40%; neurites: 40–75%) (Motta et al., 2019; Shapson-Coe et al., 2024). As a result, the substrates contained a larger extracellular space than typically reported in *ex vivo* fixed tissue, which constitutes a limitation of our simulations. Nonetheless, it is noteworthy that the neurite fraction estimations of NEXI were reasonably accurate across all substrates and configurations, and that the ground-truth values achieved in our synthetic substrates match, in fact, the NEXI estimates of *f* in the GM *in vivo* (for rat and human) (Chan et al., 2025; Jelescu et al., 2022; Uhl et al., 2025). At the same time, previous *ex vivo* or *postmortem* measurements of *f* using NEXI or SMEX yielded much higher *f* estimates, compatible with fixed tissue histology or electron microscopy analyses (Hertanu et al., 2023; Olesen et al., 2022). It is therefore plausible that our synthetic substrates are well representative of *in vivo* GM tissue.

Another limitation of the simulated substrates is that the somas are disconnected from the neurites and the neurites do not branch, whereas both features are characteristic of real GM cellular architecture. Since these features are not included in the simulations, they may further influence the estimation of NEXI parameters.

Overall, there is a need for improved numerical substrate generation frameworks capable of packing neurites more densely while allowing realistic tuning of GM cellular morphology (Aird-Rossiter, Şimşek, et al., 2026; Aird-Rossiter, Zhang, et al., 2026; De Riedmatten et al., 2024; Villarreal-Haro et al., 2023).

Despite these limitations, we were able to generate substrates that approximate key aspects of GM microstructure: they exhibit high orientation dispersion, reasonably packed neurites, beading and undulation, tunable permeability and, in one case, inclusion of somas. Although not fully realistic, these substrates are sufficient for evaluating NEXI, as they capture the main relevant GM features.

### 4.2 NEXI performance in numerical substrates

Neurites with beading and undulation introduced some time dependence in MD, which was more pronounced with stronger undulation (ϵ=0.4 , und = 1.30) (Fig. 3b, d). This behavior suggests a contribution from *structural disorder* (Novikov et al., 2014) within this diffusion-time range (15–38 ms). For these diffusion times, the characteristic longitudinal diffusion length, $\ell_{∥}\sim \sqrt{2D_{i}t}$ , is on the order of 8–12μm . This length scale is much larger than the neurite radius (~1μm ), and is therefore insensitive to neurite radius. In contrast, it is comparable to the characteristic length scale of neurite undulations and beading (ℓ ~ 10μm ), as reported for substrates with comparable amplitude (A=0.3 ) and undulation (ϵ=0.4 ) (Nguyen-Duc, Brammerloh, et al., 2026). This indicates sensitivity to along-neurite structural variations within this diffusion-time range. Thus, the weak but detectable time-dependence of MD we report in substrates with high undulation and beading likely stems from the neurite irregularities (beading, some tortuosity induced by the contextual growth) or from the extracellular space, and not from basic restriction. In the presence of somas, however, the time dependence of MD was much more pronounced. Given their size (peak radius of 7 μm), this suggests that geometric restriction alone is sufficient to induce non-Gaussian diffusion within this compartment.

Structural disorder, manifested as diffusivity time dependence, violates a key assumption of the Kärger model (Gaussian diffusion in each compartment, thus diffusivity time independence per compartment) and could therefore challenge the validity of NEXI. Although beading and undulation introduce such disorder, the resulting MD time dependence remained relatively weak across the investigated range, suggesting only limited departures from the Kärger assumptions. Consequently, NEXI estimates remained consistent with the ground-truth even under such conditions (Fig. 3a, c).

Given these relatively modest deviations from the Kärger assumptions, the observed MK time dependence is unlikely to reflect structural disorder alone. Indeed, MK, whose time dependence has been shown to arise from both structural disorder and intercompartmental exchange (Fieremans et al., 2010; H.-H. Lee et al., 2020; Novikov et al., 2014), was strongly modulated by permeability (Fig. 2b, d) and less by neurite irregularities such as beading (Fig. 3b).

Overall, these results suggest that exchange remains a dominant contributor to the observed MK time-dependence despite neurite irregularities across the investigated substrates.

However, to further assess the confounding effect of structural disorder, we generated substrates with high levels of beading and undulation (Supplementary Fig. S5). Compared with the default substrate, the substrate with higher levels of beading and undulation, and thus greater structural disorder, exhibited a higher bias in exchange time, particularly at lower permeability, showing that the model becomes increasingly sensitive to structural disorder when the contribution of membrane exchange is small.

#### 4.2.1 Effect of permeability, beading and undulation

We then examined the effect of membrane permeability in the most realistic default substrate. Overall, NEXI estimates remained robust over the examined range of permeabilities. In particular, the accuracy of extracellular diffusivity and exchange time remained fairly stable across permeability levels. However, NEXI estimates of intracellular diffusivity increased while estimates of neurite volume fraction decreased as permeability increased (Fig. 2a, c). This is likely because, at high permeability (when exchange across the membrane is larger), there are fewer intracellular molecules that have a unidirectional diffusion signature (they also experience extracellular space with 3D mobility). Thus there is seemingly a reduced proportion of water confined in unidirectional sticks but whose diffusivity is closer to the free diffusivity as they do not encounter any membrane restriction.

In terms of relative variations across permeability values, the NEXI-estimated exchange time followed the ground-truth trend. However, in terms of accuracy, the estimated exchange time showed a bias (+14% to +26% across permeability values), although lower than the bias of exchange times estimated using the MK time dependence (Figs. 2–3).

At membrane permeability κ=20μm/s , NEXI exchange time was overestimated by ~15%, with this bias remaining fairly stable across all beading levels (on average ~13%). This suggests that NEXI may remain applicable in conditions with elevated neurite beading, such as cytotoxic edema and, in particular, ischemic stroke (Budde & Frank, 2010), while still allowing reliable estimation of exchange times. However, it should be noted that cytotoxic edema is also associated with extracellular space shrinkage (Rosenblum, 2007) and with potential changes in membrane permeability due to energetic failure of ion pumps, both of which were not modeled in the present simulations but may additionally influence the diffusion signal. Increasing undulation, on the other hand, improved the accuracy of the estimated exchange time. This improvement may be partly explained by shorter ground-truth exchange time (arising from an increased membrane surface-to-volume ratio resulting from the thinner radius achieved in the substrates with higher undulations), which was shown by our permeability analysis to have slightly lower estimation bias (Fig. 2). Due to this coupling between undulation and resulting neurite radius in the substrate generation procedure, it is however challenging to conclude on the isolated impact of undulation on NEXI estimates.

#### 4.2.2 Effect of orientation dispersion

In the two configurations evaluated, the less dispersed neurite configuration ($p_{2}\;=0.69$ ) yielded a higher bias in the estimation of *t_ex_* and *D_i_* than the more dispersed configuration (*p*_2_ = 0.20) (Fig. 4a). However *f* was more overestimated in the substrate with high orientation dispersion. As only two discrete dispersion configurations were evaluated, these results do not establish a continuous dependence of estimation bias on orientation dispersion. Furthermore, the second orientation configuration tested (*p*_2_ = 0.69) exhibited substantially higher *p*_2_ values than those typically reported for GM (Lampinen et al., 2020; Salo et al., 2021).

#### 4.2.3 Effect of acquisition protocol

The clinical protocol, characterized by longer gradient pulses, longer diffusion times, and lower b-values, produced estimates of *t_ex_* and *f* that were overall remarkably comparable to those obtained with the preclinical protocol, in spite of difference of diffusion time coverage (15 - 38 ms; vs 28 - 65 ms). However, the bias in compartment diffusivities was different between the two protocols, with *D_i_* being gradually underestimated vs overestimated in the clinical vs preclinical protocol, with increasing permeability. The bias of *D_e_* was also more pronounced at high permeability for the clinical protocol.

#### 4.2.4 Effect of somas

Finally, *t_ex_* was largely overestimated (+68%) when 5% somas were included, indicating that neglecting soma compartments can affect exchange-time estimation in these substrates. It is worth pointing out that the simulated signal still exhibited a residual contribution of restriction (higher signal at longer diffusion times), particularly at low *b*-values (Supplementary Fig. S4), rather than the predominantly exchange-driven diffusion-time dependence observed experimentally *in vivo* (lower signal at longer diffusion times) (Jelescu et al., 2022; Uhl et al., 2024). The large effect of restriction in substrates that include somas is also reflected in the more pronounced MD time dependence. While the chosen soma size and relative soma-to-neurite ratio are realistic, the impact of somas may be somewhat amplified in our simulations compared to GM tissue by their disconnected geometry.

Beyond this effect on *t_ex_*, somas also influenced other parameters. The inclusion of somas increased *D_i_*, which is surprising given that, based on the stable *f* estimate, NEXI seems to impart the soma compartment to the extracellular water, as previously suggested (Jelescu et al., 2022). This over-estimation of *D_i_* could explain, in part, the high estimates found *in vivo* for this parameter (sometimes close to the free water diffusivity of 3.0 μ*m*^2^/*ms*). These estimates are reduced back to more biologically plausible values when somas are included in the model such as in SANDIX (Jallais et al., 2026; Mezzano et al., 2025). Taken together, these findings suggest that while *D_e_* and *f* remain relatively stable in the presence of somas, exchange times are more largely biased by soma. Thus, accurate estimation of exchange time may require models that also explicitly account for soma contributions, like SANDIX.

It is important to note that the simulated voxel used soma properties consistent with values obtained from microscopy-reconstructed neurons (gamma distribution for the soma radius with a 7.1μm mean and 3.6μm standard deviation (Aird-Rossiter, Zhang, et al., 2026)). However, these results are based on the limited histological data available, and regional as well as inter-species variability in soma size and volume fraction may lead to different NEXI estimates. Indeed, when the soma fraction was increased to 10%, the behavior of the synthetic signals changed quite substantially. At a given b-value, the MR signal was higher with longer diffusion times, which is a clear signature of restriction dominating over exchange. As a result, the NEXI fit for *t_ex_* failed, with the estimate hitting the upper bound. When these 10% soma were assigned the same permeability as the neurite membrane – which is not implausible biologically – the simulated MR signals retrieved their expected behavior of exchange dominating over restriction (lower signal for longer diffusion times) and an estimate for *t_ex_* was output, though overestimated at 111 ms.

This prompts a few considerations. Firstly, while 10% soma does not seem excessive in absolute terms, here this substrate had an artificially high soma-to-neurite ratio because the neurite fraction could not be increased proportionally beyond 30%. This explains why, in the case of these somas being impermeable, the signal signature changed so radically and departed also from experimental observations: in practice, exchange was always observed to dominate over disorder *in vivo* in GM (i.e., lower signal for longer diffusion times). Secondly, this also points to the fact that soma membranes are likely also permeable, and a proper GM model should account not only for neurites, soma, but also for their permeable membranes (and not just for permeable neurites) and possibly for the exchange soma-neurite. Our results suggest that permeable soma have less of a restrictive signature on the signal and could explain over-estimations of soma density or size in models of impermeable spheres such as SANDI (Palombo et al., 2020). The impact of soma on NEXI estimates should be further investigated in substrates where relative volume fractions of soma, neurites, and extracellular space are better represented, and where soma are not only permeable but also physically connected to neurites (Olesen et al., 2022).

Finally, it is noteworthy that, in most substrates and configurations, *D_i_* was quite systematically overestimated by NEXI, ranging close to the free intracellular diffusivity value (2.0 μ*m*^2^/*ms* in our simulation) but larger by ~12% than the ground-truth value obtained from the trajectories. The trajectory-based estimate is lower than the free diffusivity value as it accounts for the neurite irregularities. This is again consistent with the fact that *in vivo* estimates for *D_i_* are overall quite high (Jallais et al., 2026; Jelescu et al., 2022; Uhl et al., 2025), and should perhaps be interpreted through the prism of this known over-estimation.

### 4.3 Noise

Previous studies have already shown that finite SNR can be an important source of bias and variability in NEXI parameter estimates (Jelescu et al., 2022; Uhl et al., 2024). Here, rather than systematically characterizing the effects of noise, we evaluated its impact in a substrate configuration that incorporates features of GM microstructure absent from previous simulation studies. The goal was to assess whether the trends observed in the noiseless simulations remain detectable under realistic SNR conditions and to place the bias arising from substrate complexity in the context of the bias introduced by finite SNR.

In the presence of moderate noise (SNR = 50, typically exceeded in preclinical acquisitions and within the range commonly observed in GM in clinical acquisitions) both the variability and bias of NEXI estimates increased (Fig. 2c). The increased variability in *t_ex_* at lower permeabilities reflects the limited range of diffusion times used (15–38 ms). Compared to the noise-free case, the overall trends of parameter values were nevertheless preserved. At lower SNR (SNR = 20, Supplementary Fig. S3) the variability and bias increased substantially. These results suggest that NEXI remains reliable at moderate SNR levels, which are realistic for cortical SNR in clinical acquisitions, but becomes less reliable as SNR decreases. Overall, we demonstrated the robustness of NEXI under realistic noise conditions.

Similarly, the diffusion kurtosis metrics MD and MK exhibited not only substantial variability but also a bias compared to the noise-free case, with MD systematically higher under noisy conditions. The inaccuracy of MK estimators has been reported previously under conditions of low SNR or limited sampling directions (Veraart et al., 2011, 2013).

In the noiseless case, MK exhibited a marked dependence on diffusion time, whereas MD showed only a weak trend. At SNR = 50, the time dependence of MD became highly variable but MK slopes remained statistically different from zero and their trend across permeabilities was preserved. At SNR = 20 though, the time dependence of both MD and MK became unstable and departed from expected physical behavior due to noise.

Such noisy time dependence, particularly for MD and also observed *in vivo* (H.-H. Lee et al., 2020), may explain the difficulty in identifying clear and consistent time-dependent trends in experimental GM data, and may contribute to the discrepant findings across studies. Indeed, some reports show weak MD time dependence (Jelescu et al., 2022; H.-H. Lee et al., 2020), typically interpreted as a lack of structural disorder, and together with marked MK time dependence is consistent with the presence of inter-compartmental exchange as the dominant mechanism in GM. Others, however, report a non-negligible MD time dependence, which violates the Kärger model assumptions, implying that exchange times cannot be reliably estimated under such conditions (Mougel et al., 2024). Our study shows that realistic levels of neurite irregularities, in the form of varying degrees of beading and undulation, do introduce diffusion time-dependence. However, this time-dependence due to structural disorder is weak enough to be obscured by noise, and, even in the noiseless case, did not interfere with acceptable and meaningful estimates of NEXI parameters.

### 4.4 Future perspectives

In this work, the exchange effects simulated reflected only membrane-mediated exchange between the intra- and extracellular compartments; however, other exchange mechanisms exist. As discussed above, dendritic spines can give rise to exchange-like diffusion signatures, but their contribution is expected to be limited under the diffusion-time regimes considered here (Chakwizira et al., 2026; Şimşek et al., 2025).

Other potential exchange effects include water exchange between somas and neurites, and between connected neurite branches (Ianus et al., 2021). These mechanisms could influence the interpretation of NEXI parameters in GM, as the estimated exchange time may reflect a combination of permeative and geometric exchange processes.

Therefore, future work should rely on numerical substrates that better reproduce GM overall, with higher packing and incorporating additional GM microstructural features. Moreover, distinct membrane permeabilities could be assigned to different cellular populations (e.g., myelinated vs unmyelinated neurites, glia), a capability already available in CATERPillar, to enable a more realistic assessment of NEXI.

## 5 Conclusions

Here, we evaluated the NEXI model with numerical substrates approximating key features of GM microstructure, namely membrane permeability, neurite undulation, beading, and the presence of somas. The latter three are not explicitly accounted for in NEXI. A discrete set of substrates was generated by separately varying neurite beading, neurite undulation, orientation dispersion (using two dispersion configurations), soma inclusion, and membrane permeability. We showed that NEXI captured trends of exchange-time across permeability levels. It was also able to disentangle exchange effects from other microstructural features, although exchange time and intracellular diffusivity estimates were biased when the substrate deviated from the model assumptions, particularly in the presence of somas. Future work should aim to further improve substrate realism to better represent the complexity of neuronal morphology.

## Supplementary Material

Supplementary Material

## Data Availability

NEXI code is publicly available at https://github.com/Mic-map/graymatter_swissknife, and the CATERPillar code is available at https://github.com/Mic-map/CATERPillar. Synthetic data are available upon request.
