# Supplementary material for: Validating the neurite exchange imaging (NEXI) model of gray matter using Monte Carlo simulations in numerical substrates
Source: Imaging Neurosci (Camb). 2026 Oct 6;4:IMAG.a.1381. doi: 10.1162/IMAG.a.1381 (PMC13644052; doi:10.1162/IMAG.a.1381)
Supplement: Supplementary file 1 [file IMAG.a.1381_supp.pdf]

Validating the Neurite EXchange Imaging (NEXI) model of gray matter using Monte Carlo simulations in numerical substrates

Rita Oliveira,[1*] Jasmine Nguyen-Duc,[1] Malte Brammerloh,[1] Ileana Jelescu,[1]

[1]Department of Radiology, Lausanne University Hospital (CHUV) and University of Lausanne, Lausanne, Switzerland

*Correspondence: ana.veiga-de-oliveira@chuv.ch

Keywords: diffusion MRI, NEXI, biophysical model, numerical substrates

Supplementary Material

Validation of the permeability simulation framework

To ensure that the Monte Carlo simulation framework is accurately modeling permeability in complex geometries (Fieremans & Lee, 2018), we performed several sanity checks based on particle trajectories. First, membrane permeability was estimated from the residence times of walkers (Fieremans & Lee, 2018; Fieremans et al., 2010; Lee et al., 2025), according to:

$$t_{ex} = (1 - f)\tau_i, \tag{1}$$

where f is the neurite volume fraction and τ_i is the intracellular residence time, defined by:

$$\tau_i = \frac{1}{\kappa}\frac{V}{S}, \tag{2}$$

where V is the intracellular volume, and S is the membrane surface area.

We evaluated how the permeability estimated from particle trajectories evolved with simulation time and compared it with the permeability specified in the simulator.

We also evaluated the evolution of the intracellular volume fraction, estimated as the fraction of walkers residing in the intracellular compartment, as well as the number of membrane crossings between compartments (intra-to-extra and extra-to-intra). At equilibrium, the intracellular volume fraction is expected to reach a stable plateau, while the number of crossings in each direction should become approximately equal.

These analyses were performed for both the default substrate and a simpler geometry without undulation to evaluate the influence of substrate complexity.

Finally, to determine whether not being in fully equilibrium could affect NEXI parameter estimation, we repeated the simulations after introducing an additional 100 ms pad before the start of the diffusion encoding gradients and compared the resulting NEXI estimates with those obtained using the standard simulation framework.

The results can be found in Fig. S1. Consistent with the findings of Fieremans and Lee (2018), we observe that at short simulation times, the estimated permeability is very high and drops sharply to plateau at its input value. The plateau is reached around 50 ms in our case (Fig. S1a), contrasting to

100 ms in their case. This behavior arises, among other factors, from the way permeability is calculated from residence times, which are biased toward shorter values at early times since the simulations have only just started. As a result, differences in the y-axis values at early times are not directly meaningful for interpretation.

Therefore, we also examined the volume fraction of walkers in the intracellular compartment (Fig. S1b). We observe that an equilibrium is reached after approximately 50 ms, which is comparable to or longer than the diffusion range studied here. However, the deviation from the equilibrium at early times remains very small (2%). In addition, we analyzed the number of crossings between compartments (intra→extra and extra→intra) (Fig. S1c). These curves closely follow each other and reach a plateau after around 50 ms, consistent with the establishment of equilibrium. The difference between the number of crossings in each direction remains below 2.5%, indicating once more that the bias is small.

We also note that this effect is more pronounced in complex substrates (Fig. S1 left), whereas in simpler geometries (e.g., without undulation, Fig. S1 right), the discrepancy is even more minimal.

Despite these effects, NEXI estimates remain stable. To test the impact of higher apparent permeability at short times, we compared two simulations, one of which includes an additional 100 ms of padding time before the effective start of the PGSE scheme (Fig. S1g). With or without padding, the resulting NEXI estimates remain essentially unchanged.

Finally, in this work, the ground-truth exchange time was initially estimated from the substrate geometry using the Equations 1-2 , which relies on the barrier-limited exchange assumption. To assess the validity of this approximation, we compared those values with exchange times estimated directly from particle trajectories and found close agreement between the two approaches (Fig. S1h). We therefore kept the substrate-based estimates throughout the study.

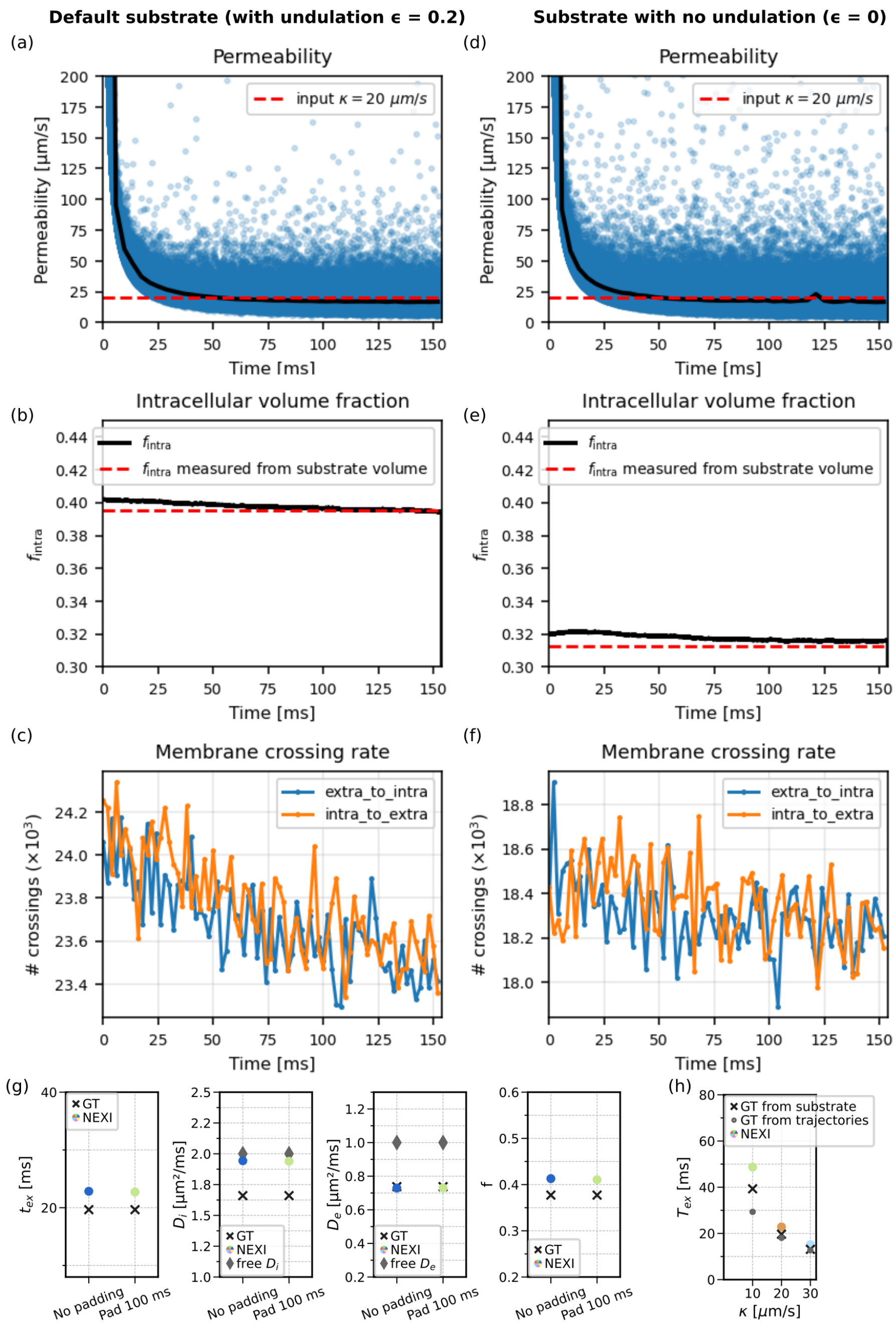

Default substrate (with undulation ϵ = 0.2)
Substrate with no undulation (ϵ = 0)
(a)
Permeability
input κ = 20 μm/s
Permeability [μm/s]
Time [ms]
(d)
Permeability
input κ = 20 μm/s
Permeability [μm/s]
Time [ms]
(b)
Intracellular volume fraction
f_intra
f_intra measured from substrate volume
Time [ms]
(e)
Intracellular volume fraction
f_intra
f_intra measured from substrate volume
Time [ms]
(c)
Membrane crossing rate
extra_to_intra
intra_to_extra
crossings (×10³)
Time [ms]
(f)
Membrane crossing rate
extra_to_intra
intra_to_extra
crossings (×10³)
Time [ms]
(g)
GT
NEXI
t_ex [ms]
D_i [μm²/ms]
free D_i
D_e [μm²/ms]
free D_e
f
No padding
Pad 100 ms
(h)
GT from substrate
GT from trajectories
NEXI
T_ex [ms]
κ [μm/s]

Figure S1: **Validation of the permeability simulation framework.** (a) Membrane permeability estimated from the intracellular residence time of walkers, calculated from particle trajectories, for the default substrate. Blue dots represent individual walker estimates, the black curve the average, and the red dashed line the input permeability in the simulator ($\kappa = 20$ μm/s). We observe that at shorter simulation times the estimated permeability is higher than at later times, where it reaches a plateau around 50 ms. (b) Time evolution of the intracellular volume fraction estimated from particle trajectories (black), compared with the value measured directly from the substrate geometry (red dashed line). We observe that an equilibrium is reached after approximately 50 ms, which in face value seems higher compared to the diffusion range at study here. However, the deviation from the equilibrium at early times remains very small ($\sim$2%). (c) Number of membrane crossings per time interval for particles moving between between compartments (intra to extra and extra to intra). These curves closely follow each other and reach a plateau after around 50 ms, consistent with the previous results. The difference between the number of crossings in each direction remains below 2.5%, indicating once more that the bias is small. We note that this effect is more pronounced in complex substrates (a–c), whereas in simpler geometries, e.g., without undulation (d–f), the discrepancy is even smaller. (g) To ensure that equilibrium was reached in the simulation framework before applying the diffusion gradient pulses, we introduced an additional delay of 100 ms prior to gradient application ("Pad 100 ms") and compared the resulting estimates with those obtained using the standard framework ("No padding"), where gradients were applied immediately. The resulting NEXI estimates remained essentially unchanged. Black cross markers indicate ground-truth (GT) values and gray diamond markers indicate D_i and D_e, the intrinsic/free diffusivities specified in the simulator. (h) NEXI-estimated exchange times for simulations performed with different membrane permeabilities κ, together with the ground-truth estimated i) from the substrate geometry using the relationship between membrane permeability and neurite surface-to-volume ratio (black crosses) and ii) from particle trajectories (gray circles). Both ground-truth estimation approaches were in close agreement (differing by approximately 2%).

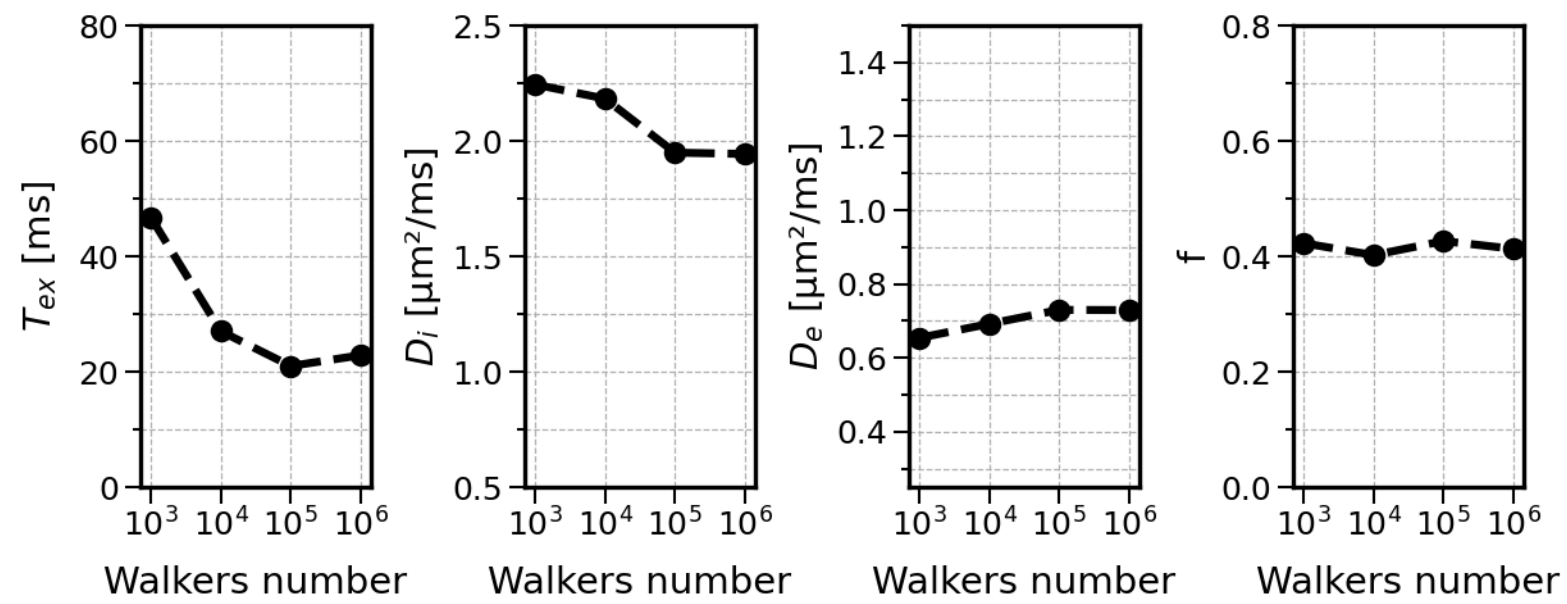

Figure S2: **NEXI estimates as a function of the number of walkers simulated.** Increasing the number of walkers beyond 10^5 did not substantially improve the estimates, as most parameters reached a plateau.

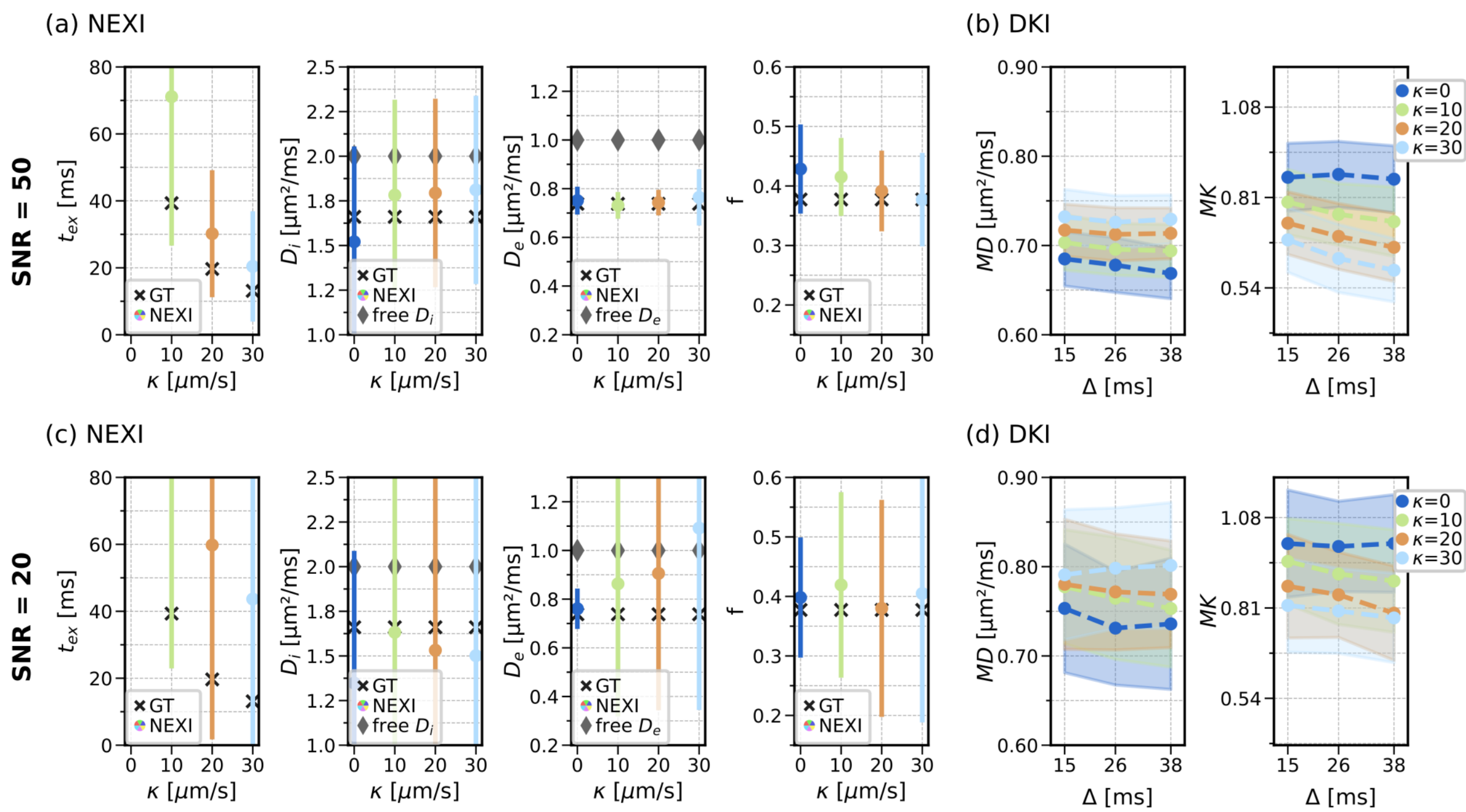

Figure S3: **NEXI performance across permeability levels with SNR = 50 and 20.** NEXI (a, c) and DKI (b, d) estimates with added noise at SNR = 50 and SNR = 20, respectively. With decreasing SNR, NEXI estimates show higher variability and increased bias. The time dependence of MD and MK becomes unstable and departs from expected physical behavior at lower SNR levels. Black cross markers indicate ground-truth (GT) values and gray diamond markers indicate D_i and D_e, the intrinsic/free diffusivities specified in the simulator. The error bars represent the standard deviation of the estimates obtained from 500 noise realizations added to the noiseless Monte Carlo signal.

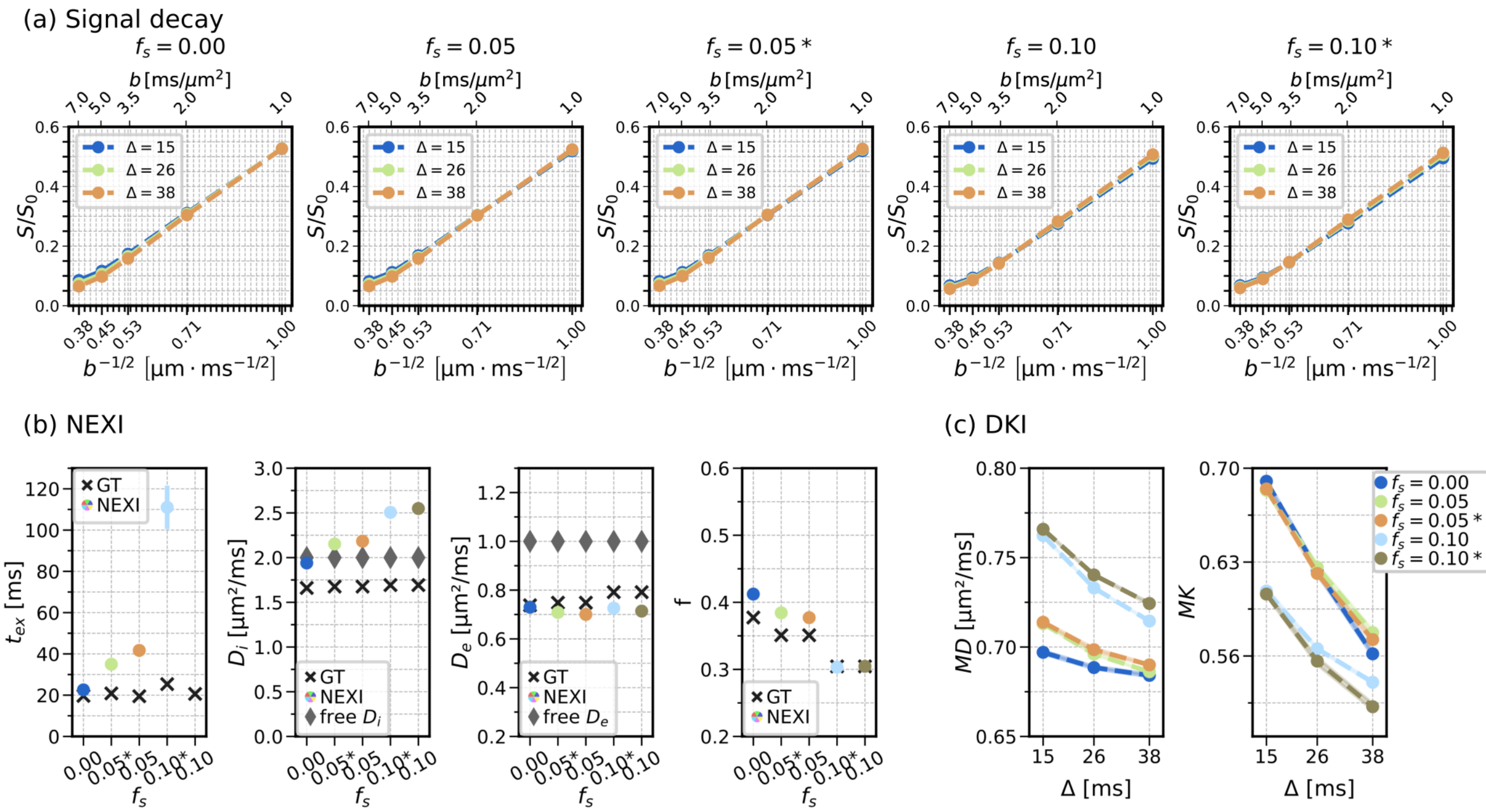

Figure S4: **NEXI performance in the presence of 5% and 10% somas.** We evaluated the following conditions: $f_s = 0$; $f_s = 0.05$ impermeable somas; $f_s = 0.05$ with soma–extracellular exchange at $20\ \mu\text{m/s}$; $f_s = 0.10$ impermeable somas; and $f_s = 0.10$ with soma–extracellular exchange at $20\ \mu\text{m/s}$. The asterisk (*) indicates cases where soma–extracellular exchange is included, whereas the remaining cases assume impermeable somas. All cases used neurite membrane permeability of $20\ \mu\text{m/s}$. Signal decays for these conditions are shown in (a). For the case of impermeable somas, as the soma fraction increased, the soma contribution progressively dominated the overall signal, and the diffusion MRI signal no longer reproduced the *in vivo* observation of decreasing signal with increasing diffusion time (Jelescu et al., 2022; Olesen et al., 2022), preventing estimation of an exchange time (b). Introducing soma–extracellular exchange partially restored the *in vivo*-like signal behavior. However, with a 10% soma volume fraction, the resulting t_{ex} estimates still exhibited substantial bias and variability. In contrast, the case of 5% soma volume fraction produced a signal decay that more closely matched the expected *in vivo* behavior (a) and yielded a more accurate t_{ex} estimate (b), despite still a bias of $+68\%$. The DKI metrics showed similar time dependence in the exchange and no-exchange cases, although MD increased and MK decreased for higher soma volume fractions due to the greater contribution of less restricted spherical diffusion (c). Black cross markers indicate ground-truth (GT) values and gray diamond markers indicate D_i and D_e, the intrinsic/free diffusivities specified in the simulator. The error bars represent the standard deviation of the estimates obtained from the 3 independent runs of the Monte Carlo simulations.

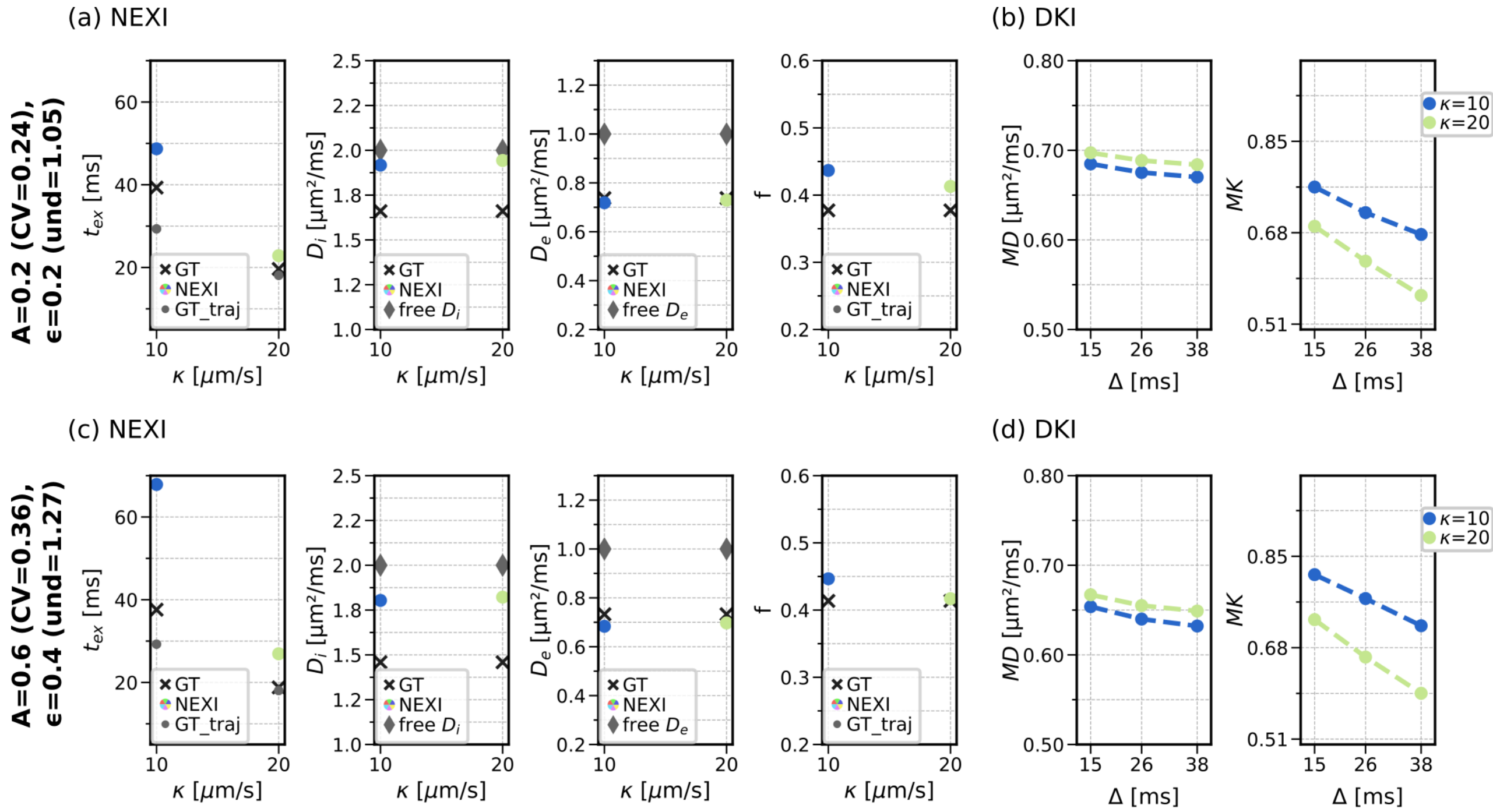

Figure S5: **Effect of structural disorder and neurite volume fraction.** A highly disordered substrate ($\epsilon = 0.4$, $A = 0.6$) was compared to the default substrate ($\epsilon = 0.2$, $A = 0.2$). Results are shown for membrane permeabilities $\kappa = 10$ and 20 μm/s (a,c). Exchange time was more overestimated at low permeabilities and increasing structural disorder further reduced the accuracy of the exchange time estimates (at $\kappa = 10$ μm/s, the error increased from 26% in the default substrate (a) to 79% in the more disordered substrate). Black cross markers indicate ground-truth (GT) values (for t_{ex} we show the GT computed from the substrate geometry using the relationship between membrane permeability and neurite surface-to-volume ratio (black crosses) and computed from the particle trajectories (gray circles)); and gray diamond markers indicate D_i and D_e, the intrinsic/free diffusivities specified in the simulator.

References

Fieremans, E., & Lee, H.-H. (2018). Physical and numerical phantoms for the validation of brain microstructural MRI: A cookbook. *NeuroImage*, *182*, 39–61. https://doi.org/10.1016/j.neuroimage.2018.06.046

Fieremans, E., Novikov, D. S., Jensen, J. H., & Helpern, J. A. (2010). Monte Carlo study of a two-compartment exchange model of diffusion. *NMR in Biomedicine*, *23*(7), 711–724. https://doi.org/10.1002/nbm.1577

Jelescu, I. O., De Skowronski, A., Geffroy, F., Palombo, M., & Novikov, D. S. (2022). Neurite Exchange Imaging (NEXI): A minimal model of diffusion in gray matter with inter-compartment water exchange. *NeuroImage*, *256*, 119277. https://doi.org/10.1016/j.neuroimage.2022.119277

Lee, H.-H., Novikov, D. S., Fieremans, E., & Huang, S. Y. (2025). Revealing membrane integrity and cell size from diffusion kurtosis time dependence. *Magnetic Resonance in Medicine*, *93*(3), 1329–1347. https://doi.org/10.1002/mrm.30335

Olesen, J. L., Østergaard, L., Shemesh, N., & Jespersen, S. N. (2022). Diffusion time dependence, power-law scaling, and exchange in gray matter. *NeuroImage*, *251*, 118976. https://doi.org/10.1016/j.neuroimage.2022.118976
